# Enhanced Selenium Supplement Extends Lifespan and Delays Multi‐Organs Aging by Regulating the Sik1 Pathway Through Maintaining Calcium Homeostasis

**DOI:** 10.1002/advs.202511813

**Published:** 2025-10-02

**Authors:** Yang Yu, Jintao Song, Mengjiao Guo, RuZe Ma, Mingyang Du, Zhe Xun, Xu Liu, RongXia Xu, Xiaochun Xie, Peilin Qi, Yujie Chen, Dan Shao, Chao Yang, Liang Wang, Xiaoyu Song, Difei Wang

**Affiliations:** ^1^ Health Sciences Institute China Medical University Shenyang Liaoning 110122 China; ^2^ College of Basic Medical Science Key Laboratory of Medical Cell Biology Ministry of Education Key Laboratory of Liaoning Province China Medical University Shenyang Liaoning 110122 China; ^3^ Department of Gerontology Shengjing Hospital of China Medical University Shenyang Liaoning 110004 China; ^4^ National Engineering Research Center for Tissue Restoration and Reconstruction South China University of Technology Guangzhou Guangdong 510006 China; ^5^ Department of Orthopedics Center for Orthopedic Surgery The Third Affiliated Hospital of Southern Medical University Guangzhou 510630 China

**Keywords:** aging, calcium, nanoparticle, selenium, Sik1

## Abstract

Selenium supplementation has potential in treating aging‐related disorders like neurodegenerative and cardiovascular diseases, but its use is limited by poor bioavailability, a narrow therapeutic window, and unclear mechanisms. To overcome this, redox‐dual‐responsive diselenide‐bridged mesoporous silica nanoparticles (SeMSNs) are developed. SeMSNs effectively reduce oxidative stress and downregulate senescence markers (p16, p21), suppressing senescence in both naturally aged primary mouse embryonic fibroblasts (MEFs) and H_2_O_2_‐induced HEK‐293T cells. They show prolonged antioxidant effects (*p* < 0.05) and lower cytotoxicity (*p* < 0.01) than commercial selenomethionine. In aged mice, SeMSNs extend lifespan, reduce frailty, and improve age‐related conditions, including muscle atrophy, renal dysfunction, cognitive decline, and hepatic steatosis, while restoring metabolic balance. They outperform conventional organically‐bridged mesoporous silica nanoparticles (MSNs) and disulfide‐bridged MSNs (SMSNs) (*p* < 0.01). Mechanistically, SeMSNs upregulate selenoproteins (GPx1, SelK), suppress endoplasmic reticulum (ER) stress‐mediated calcium release, maintain calcium homeostasis, and inhibit NFATc2‐driven Sik1 transcription, reducing p21/p16. Clinical data confirm an inverse correlation between selenium levels and aging biomarkers (*p* < 0.0001). SeMSNs also restore adipogenic differentiation in human adipose progenitor cells via calcium‐NFATc2‐Sik1 signaling. These results demonstrate the superiority of SeMSNs over traditional selenium forms, providing a nanotherapeutic strategy to combat multi‐organ aging and promote healthy longevity.

## Graphical abstract

Aging involves physiological decline and heightened disease susceptibility, necessitating effective interventions. Redox‐responsive selenium‐loaded nanoparticles (SeMSNs) are developed, which delay cellular senescence by reducing oxidative stress and senescence markers (p16/p21). In aged mice, SeMSNs extend lifespan, improve organ function, and restore metabolic homeostasis by targeting the calcium‐NFATc2‐Sik1 pathway via selenoprotein upregulation. Clinically, selenium levels inversely correlate with aging biomarkers, highlighting SeMSNs’ potential as a multi‐organs anti‐aging therapy.

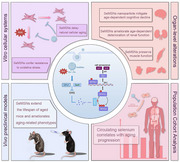

## 1 Introduction

Aging is a multifaceted biological process marked by the progressive deterioration of physiological functions, impaired homeostatic regulation, and the accumulation of molecular, cellular, and tissue damage over time. Key characteristics include genomic instability, telomere attrition, epigenetic changes, endoplasmic reticulum stress (ERS), mitochondrial dysfunction, and cellular senescence.^[^ ^1^, ^2^, ^3^, ^4^ ^]^ By the latter half of the 2070s, the global population aged ≥ 65 years is projected to exceed 2.2 billion, surpassing the number of individuals under 18 for the first time in history.^[^ ^5^ ^]^ Multimorbidity is especially prevalent in the elderly, with over 60% of older adults affected by two or more chronic diseases, and more than half managing three or more simultaneously.^[^ ^6^, ^7^ ^]^ In this context, understanding the molecular mechanisms behind aging‐related multimorbidity and developing targeted interventions are essential for achieving the three main objectives of anti‐aging research: extending lifespan, slowing aging progression, and enhancing healthspan. Advances in this field will directly address the medical challenges posed by an aging population and provide a scientific foundation for improving the quality of life in older adults.

In healthy aging strategies, nutritional supplements synergize with optimized dietary and lifestyle interventions by modulating aging‐related molecular pathways.^[^ ^8^, ^9^ ^]^ Notably, NMN exerts multi‐organ anti‐aging effects by elevating NAD^+^ levels to activate the SIRT1 pathway, thereby significantly enhancing mitochondrial function while reducing oxidative stress and DNA damage.^[^ ^10^ ^]^ Similarly, curcumin delays aging and related diseases through pleiotropic mechanisms involving oxidative stress regulation, anti‐inflammatory actions, telomere maintenance, and sirtuin protein modulation.^[^ ^11^ ^]^ However, practical applications face significant challenges: bioactive compounds like resveratrol and curcumin suffer from limited bioavailability due to poor aqueous solubility and first‐pass metabolism, while excessive supplementation of antioxidants such as vitamins C/E may disrupt reactive oxygen species (ROS) signaling homeostasis, potentially inducing cellular toxicity or even increasing hemorrhagic risk.^[^ ^12^, ^13^, ^14^ ^]^ Future development of anti‐aging supplements should focus on: 1) innovative formulation strategies to enhance bioavailability; 2) optimized dosing regimens to minimize toxicity; and 3) long‐term clinical studies to validate efficacy.

Selenium, an essential trace element with diverse biological activities, plays a critical role in healthy aging.^[^ ^15^, ^16^, ^17^ ^]^ ≈1 billion people worldwide are affected by selenium deficiency, which is closely linked to neurological disorders, cardiovascular abnormalities, malignancies, and immune dysfunction.^[^ ^18^, ^19^, ^20^ ^]^ Substantial evidence supports the anti‐aging effects of selenium through multiple mechanisms: 1) Selenomethionine (SeMet) effectively suppresses Fe^2+^/H_2_O_2_‐ or Aβ‐induced free radical generation, demonstrating therapeutic potential for Alzheimer's disease characterized by oxidative stress;^[^ ^21^ ^]^ 2) Selenium supplementation elevates serum GPx3 levels, a selenoprotein predominantly localized in the basement membrane of renal proximal tubules, modulating mitochondrial quality control pathways to mitigate heavy metal‐induced renal aging;^[^ ^22^ ^]^ and 3) Our recent findings reveal that selenium supplementation significantly attenuates age‐related muscle atrophy by preserving redox homeostasis and regulating muscle protein metabolism.^[^ ^23^ ^]^ Recent clinical trials in patients with advanced non‐small cell lung cancer (NSCLC) demonstrated that oral administration of selenium nanoparticles (SeNPs) as a dietary supplement (200 µg day^−1^) in combination with Bev+AP chemotherapy significantly enhanced therapeutic outcomes compared to chemotherapy alone. The SeNPs combination group showed remarkable tumor regression, with progression disease rates decreasing dramatically from 50% to 0% and partial response rates increasing to 83.3%, along with significantly improved objective response rate and disease control rate.^[^ ^24^ ^]^ Importantly, this regimen maintained excellent safety profiles without triggering fluctuations in pro‐inflammatory or immunosuppressive cytokines. These compelling findings not only establish SeNPs as a safe and effective adjuvant therapy for advanced NSCLC but also provide valuable clinical translation data for nano‐selenium formulations in oncology. Despite selenium's proven benefits in reducing oxidative damage, maintaining genomic stability, and delaying telomere shortening, its narrow therapeutic window, limited bioavailability, and specific mechanisms in multi‐organ protection during natural aging require further investigation.

Nanodelivery carriers have emerged as a next‐generation platform for gene and drug delivery, offering tunable physicochemical properties such as size, composition, and surface modifications.^[^ ^25^ ^]^ Our team has developed organically‐bridged mesoporous silica nanoparticles (MSNs) by incorporating functional diselenide bonds into the silica framework at the molecular level, addressing the critical challenge of poor biodegradability in conventional silica materials.^[^ ^26^ ^]^ This nanocarrier exhibits unique dual redox‐responsive properties, allowing for more precise maintenance of redox homeostasis compared to traditional antioxidants, aligning with the core goal of preserving organismal homeostasis in anti‐aging research. Building on this breakthrough, a comprehensive research framework was established: first, this study constructed a natural aging mouse model with MSNs, disulfide‐bridged MSNs (SMSNs), commercially available SeMet as controls and then compared the effects of diselenide‐bridged MSNs (SeMSNs) on lifespan extension, frailty delay, and multi‐organ anti‐aging. Next, key pathways and targets were identified through multi‐organ transcriptome sequencing, followed by in‐depth mechanistic studies. Finally, clinical translation was integrated by analyzing the correlation between serum selenium levels and aging biomarkers in the elderly, and validating the clinical effects of SeMSNs using primary adipose precursor cells (APCs) models. This systematic approach provides a solid theoretical foundation and clinical evidence for the application of nano‐selenium in anti‐aging research.

## 2 Results

### 2.1 SeMSNs Significantly Delay Cellular Senescence

**Figure 1:**
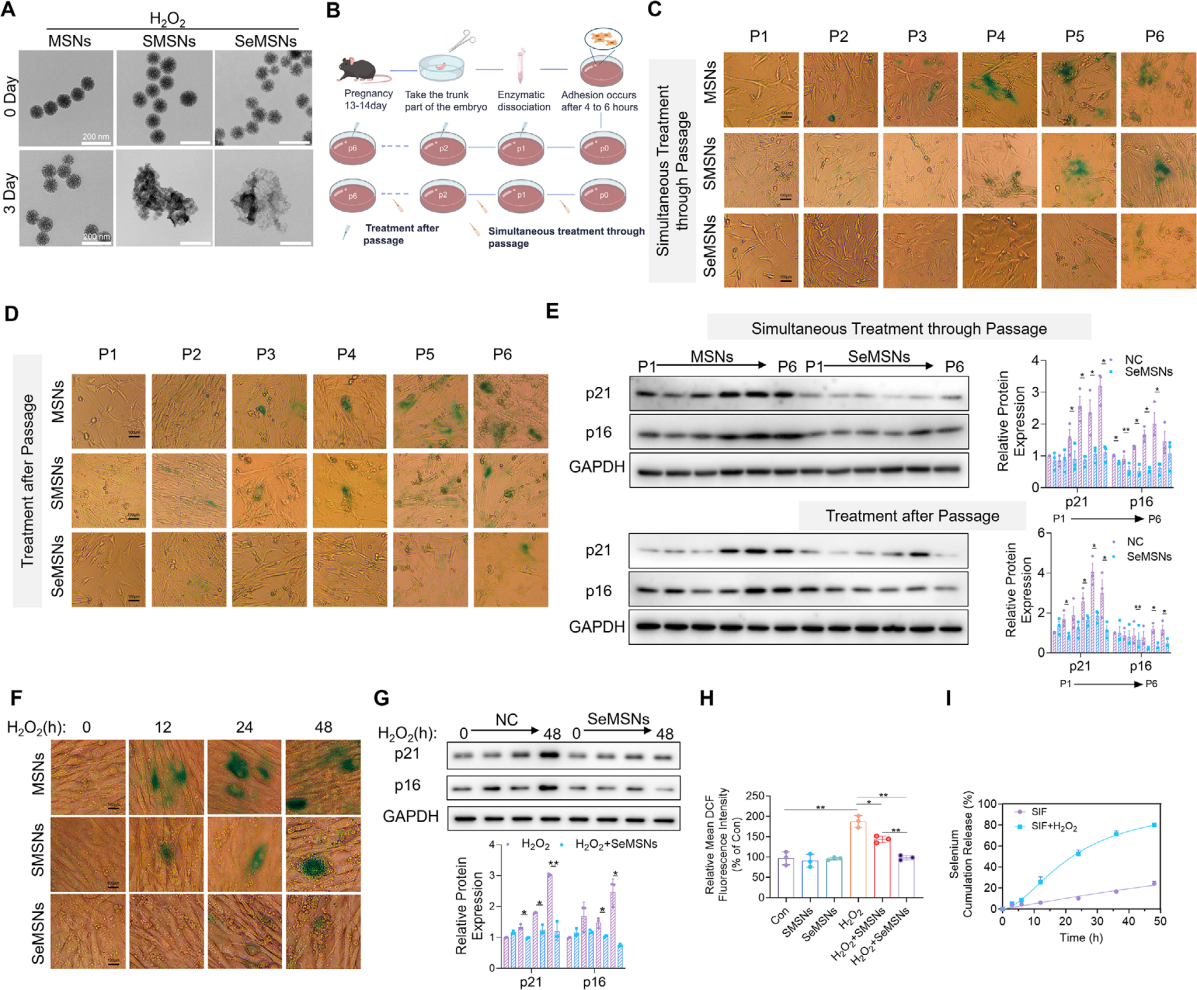
SeMSNs significantly delay cellular senescence. A) Electron microscopy comparison of MSNs, SMSNs, and SeMSNs nanoparticles in simulated intestinal fluid on 0 and 3 Days, showing structural degradation. (*n* = 3 independent experiments). B) Schematic workflow for MEF isolation and drug administration regimens: continuous treatment (drug applied at every passage) versus single‐passage treatment (drug applied at one designated passage), monitored over six passages. C) SA‐β‐gal staining of MEFs under continuous drug treatment (applied at every passage from P1 to P6, *n* = 3 independent experiments). D) SA‐β‐gal staining of MEFs subjected to post‐passage drug treatment (drug applied at one designated passage from P1 to P6, *n* = 3 independent experiments). E) Western blot analysis of p21 and p16 expression in MEFs under continuous versus post‐passage drug treatment regimens (P1–P6, *n* = 3 independent experiments), with quantitative densitometry normalized to GAPDH. F) SA‐β‐gal staining of MEFs in three experimental groups: NC (H_2_O_2_), H_2_O_2_ + SMSNs, and H_2_O_2_ + SeMSNs, imaged at 0, 12, 24, and 48 h post‐treatment. *n* = 3 independent experiments. G) Western blot analysis of p21 and p16 expression in MEFs exposed to H_2_O_2_ under two regimens: NC (H_2_O_2_) and SeMSNs (H_2_O_2_ + SeMSNs), analyzed at 0, 12, 24, and 48 h post‐treatment, with quantitative densitometry normalized to GAPDH (*n* = 3 independent experiments). H) ROS detection via DCFH‐DA fluorescence in six groups: untreated (Con), SMSNs, SeMSNs, H_2_O_2_, H_2_O_2_ + SMSNs, and H_2_O_2_ + SeMSNs (*n* = 3 independent experiments). I) Time‐dependent selenium (Se) release from SeMSNs nanoparticles in simulated intestinal fluid (SIF) and SIF + H_2_O_2_, quantified at 0, 10, 20, 30, 40, and 50 h (*n* = 3 independent experiments). All data are presented as mean ± SD. One‐way analysis of variance and Tukey multiple comparison test were used to evaluate statistical differences. ^*^ *p* < 0.05, ^**^ *p* < 0.01.

To evaluate the anti‐aging therapeutic effects of SeMSNs, this study synthesized MSNs and SMSNs as controls, and SeMSNs following the methodology developed by our research group.^[^ ^23^, ^26^ ^]^ Particle size analysis showed that the synthesized MSNs, SMSNs, and SeMSNs had sizes of 113.9 ± 2.14, 105.8 ± 2.73, and 115.4 ± 2.03 nm, respectively (Figure S1, Supporting Information). Upon H_2_O_2_ stimulation, both SMSNs and SeMSNs exhibited significant degradation by day three, responding to the signal stimulus (**Figure** **1**A). The cytotoxicity assay results indicated that none of the various types of MSNs exhibited significant cytotoxicity at a concentration of 60 µg mL^−1^ (Figure S2A–C, Supporting Information). In the cellular ROS detection, SeMSNs demonstrated remarkable antioxidant effects compared to MSNs and SMSNs. At concentrations of 10 µg mL^−1^ and above, SeMSNs were able to restore the intracellular ROS levels induced by H_2_O_2_ stimulation back to the control group level (Figure S2D–F, Supporting Information). Therefore, we selected the concentration of 10 µg mL^−1^ SeMSNs for subsequent cellular experiments. Additionally, the effects of the nanovesicles on cellular senescence were evaluated using primary mouse embryonic fibroblasts (MEFs) subjected to serial passaging to model natural aging. Two treatment regimens were implemented: 1) a co‐culture approach, where cells were continuously maintained in 10 µg mL^−1^ nanovesicle‐containing medium to observe synchronized drug effects during passaging, and 2) a post‐passaging protocol, where cells were expanded in normal medium and treated with 10 µg mL^−1^ nanovesicles 24 h after plating to examine delayed therapeutic effects (Figure 1B). Cellular senescence was assessed through β‐galactosidase staining and analysis of senescence markers p16 and p21. The results revealed distinct patterns across treatment groups: in both protocols, MEFs treated with MSNs showed strong β‐galactosidase positivity by passage 3, whereas the SMSNs group exhibited significant staining starting at passage 5. Notably, SeMSNs treatment effectively inhibited β‐galactosidase expression throughout the experimental period (Figure 1C,D). This was further confirmed by p16 and p21 expression analysis, which showed that SeMSNs‐treated MEFs maintained stable levels of these markers from passage 1 to passage 6, in contrast to the progressive increase observed in control groups (Figure 1E). In addition to the natural aging model, a drug‐induced senescence model was established using H_2_O_2_‐stimulated HEK‐293T cells. Consistent with the previous results, SeMSNs effectively suppressed the H_2_O_2_‐induced increase in β‐galactosidase‐positive cells and the upregulation of p16 and p21 expression (Figure 1F,G). The effects of various concentrations of different types of MSNs on the expression of p16 and p21 in H_2_O_2_‐stimulated HEK‐293T cells showed that MSNs had no significant impact on the H_2_O_2_‐induced upregulation of p16 and p21. Treatment with 10 µg mL^−1^ SMSNs slightly inhibited the expression of p16 and p21, while the same concentration of SeMSNs significantly suppressed their expression (Figure S2G–I, Supporting Information). Our in vitro experiments further demonstrated that SeMSNs possessed a superior ROS scavenging ability compared to SMSNs, with a two‐fold higher efficacy (*p* < 0.01; Figure 1H). In simulated intestinal fluid (SIF), SeMSNs displayed time‐dependent release profiles under H_2_O_2_ treatment (Figure 1I). Collectively, these results indicate that SeMSNs effectively suppress cellular senescence with greater efficacy than SMSNs.

### 2.2 SeMSNs Extend the Lifespan of Aged Mice and Ameliorate Aging‐Related Phenotypes

**Figure 2:**
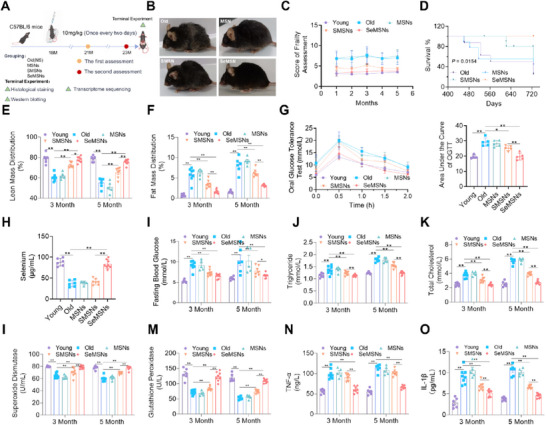
SeMSNs extend the lifespan of aged mice and ameliorate aging‐related phenotypes. A) Experimental design for establishing a natural aging mouse model and evaluating therapeutic intervention strategies. B) Appearance of mice treated with nanoparticle intervention. The appearance of mice treated with MSNs, SMSNs, and SeMSNs by gavage at 18 months of age for 5 months. C) Aging evaluation index of mice with nanoparticle intervention. Mice were divided into the young group, aging group, MSNs group, SMSNs group, and SeMSNs group. Frailty assessment parameters were evaluated monthly (*n* = 5–8 mice per group). D) Survival curve of mice treated with nanoparticles. Survival curves for the five groups of mice were plotted after 10 months of nanoparticle intervention (*n* = 10 mice per group at 10 months). Survival differences among the groups were analyzed using Kaplan–Meier survival curves, with significance evaluated by the Log‐rank test. E) Changes in muscle mass of mice at 3 and 5 months post‐nanoparticle intervention (*n* = 5–8 mice per group). F) Changes in fat mass of mice at 3 and 5 months post‐nanoparticle intervention (*n* = 5–8 mice per group). G) Oral Glucose Tolerance Test (OGTT) in mice treated with nanoparticles. After 5 months of nanoparticle intervention, OGTT was conducted on the five groups of mice, and the area under the OGTT curve was calculated (*n* = 5–8 mice per group). H) Serum selenium levels in experimental mice following 5 months of nanoparticle intervention (*n* = 5–8 mice per group). I) Fasting blood glucose levels of mice after 3 and 5 months of nanoparticle intervention (*n* = 5–8 mice per group). J) Triglyceride levels in the serum of mice at 3 and 5 months post‐nanoparticle intervention (*n* = 5–8 mice per group). K) Serum total cholesterol levels in mice following 3 and 5 months of nanoparticle intervention (*n* = 5–8 mice per group). L) Serum superoxide dismutase (SOD) levels in mice following 3 and 5 months of nanoparticle intervention (*n* = 5–8 mice per group). M) Serum glutathione peroxidase (GSH‐Px) levels in mice following 3 and 5 months of nanoparticle intervention (*n* = 5–8 mice per group). N) Serum TNF‐α levels in mice following 3 and 5 months of nanoparticle intervention (*n* = 5–8 mice per group). O) Serum IL‐1β levels in mice following 3 and 5 months of nanoparticle intervention (*n* = 5–8 mice per group). All data are presented as mean ± SEM. Survival curve analysis used the Log‐rank test; other quantitative data were analyzed using one‐way analysis of variance and the Tukey multiple comparison test to evaluate statistical differences. ^*^ *p* < 0.05, ^**^ *p* < 0.01.

The effects of MSNs, SMSNs, and SeMSNs on lifespan, frailty index, and aging phenotypes were evaluated using a natural aging mouse model. Tissue distribution analysis of selenium showed that 1 h after administration of SeMSNs, selenium was most highly enriched in the liver (*p* < 0.01), kidneys (*p* < 0.01), and spleen (*p* < 0.01). Its levels were also significantly higher in the brain (*p* < 0.05), lungs (*p* < 0.01), skeletal muscle (*p* < 0.05), and heart (*p* < 0.01) compared to the non‐administered group (Figure S3, Supporting Information). Oral administration commenced at 18 months of age (≈55 human years; **Figure** **2**A). After five months of treatment, typical senescent phenotypes, including sparse and dull fur as well as kyphosis, were observed in the aged mice. MSNs treatment showed no significant improvement, while SMSNs administration partially alleviated these aging manifestations. In contrast, SeMSNs‐treated mice maintained dense, glossy fur and did not develop kyphosis, demonstrating superior anti‐aging effects (Figure 2B). Frailty index scoring revealed that, after one month of treatment, SeMSNs‐treated aged mice exhibited frailty indices comparable to those of adult control mice, significantly lower than those in SMSNs‐treated, MSNs‐treated, and untreated old groups (Figure 2C). Furthermore, SeMSNs administration significantly extended lifespan, with all treated mice surviving beyond 24 months (Figure 2D). Body composition analysis showed SeMSNs‐specific preservation of lean mass (*p* < 0.01; Figure 2E) and attenuation of age‐associated adiposity (*p* < 0.01; Figure 2F), compared to untreated controls and other treatment groups. Metabolic profiling indicated SeMSNs‐mediated restoration of glucose homeostasis, with oral glucose tolerance test (OGTT) curves resembling those of young controls (*p* < 0.01; Figure 2G). After five months of SeMSNs supplementation, blood selenium levels in aged mice were effectively maintained at adult physiological concentrations (*p* < 0.01; Figure 2H). Fasting blood glucose (FBG) levels in SeMSNs‐treated mice remained stable throughout the intervention period, while hyperglycemia progressed in the aged control group (*p* < 0.01; Figure 2I). Serum biomarkers confirmed broad rejuvenation, including normalization of lipid profiles (triglycerides: *p* < 0.01; cholesterol: *p* < 0.01; Figure 2J,K) and enhanced antioxidant capacity (SOD: *p* < 0.01; GSH‐Px: *p* < 0.01; Figure 2L,M). Notably, SeMSNs uniquely restored circulating selenium to youthful reference ranges (*p* < 0.01 vs aged controls; Figure 2H), correlating with reduced systemic inflammation (TNF‐α: *p* < 0.01; IL‐1β: *p* < 0.01; Figure 2N,O). Overall, SeMSNs treatment significantly prolonged lifespan and attenuated senescent decline, demonstrating superior efficacy over SMSNs.

### 2.3 SeMSNs Attenuate Multi‐Organ Aging and Restore Function In Aged Mice

**Figure 3:**
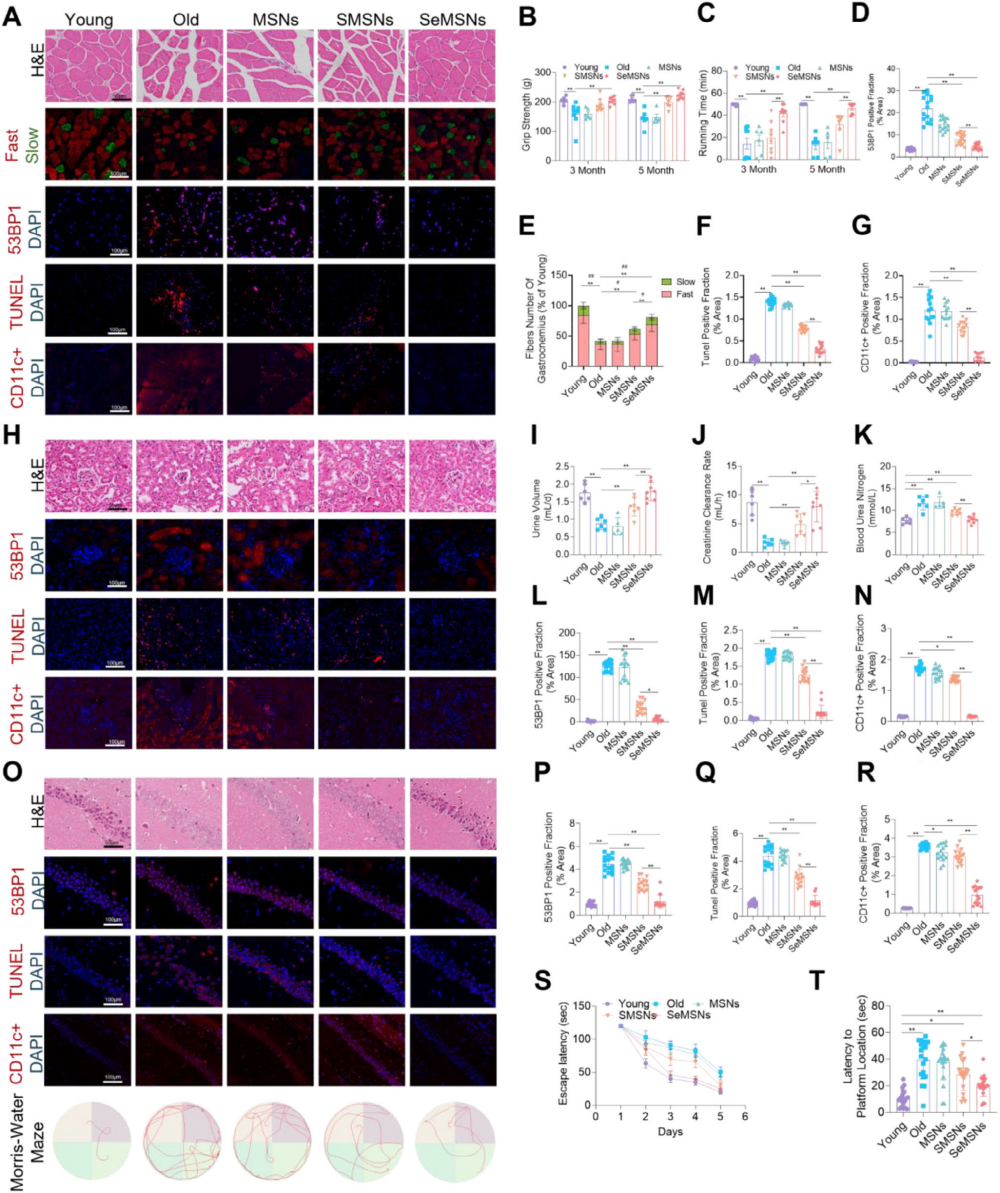
SeMSNs attenuate multi‐organ aging and restore function in aged mice. A) Multiplex histological analysis of gastrocnemius muscles from five experimental groups, including H&E staining, fast/slow myofiber immunofluorescence (MyHC I/II), 53BP1 foci, TUNEL assay, and CD11c^+^ cell infiltration. B) Grip strength of mice after 3 and 5 months of nanoparticle intervention (*n* = 5–8 mice per group). C) Continuous running time of mice after 3 and 5 months of nanoparticle intervention (*n* = 5–8 mice per group). D) Quantitative analysis of 53BP1 foci in gastrocnemius muscles across five experimental groups (5 fields per mouse were analyzed, *n* = 3 mice per group). E) Quantitative analysis of fast (MyHC II) and slow (MyHC I) myofiber composition in gastrocnemius muscles across five experimental groups (5 fields per mouse were analyzed, *n* = 3 mice per group). F) Quantitative analysis of TUNEL foci in gastrocnemius muscles across five experimental groups (5 fields per mouse were analyzed, *n* = 3 mice per group). G) Quantitative assessment of CD11c^+^ immune cell infiltration in gastrocnemius muscles across five experimental groups (5 fields per mouse were analyzed, *n* = 3 mice per group). H) Multimodal analysis of renal tissues across five experimental groups: H&E staining, 53BP1 foci, TUNEL assay, and CD11c^+^ immune infiltration. I) Daily urine output recorded in five experimental groups of mice using metabolic cages (*n* = 5–8 mice per group). J) Quantitative analysis of creatinine clearance rates in five experimental mouse groups (*n* = 5–8 mice per group). K) Quantitative analysis of blood urea nitrogen levels in five experimental mouse groups (*n* = 5–8 mice per group). L) Quantitative analysis of 53BP1 foci in kidneys across five experimental groups (5 fields per mouse were analyzed, *n* = 3 mice per group). M) Quantitative analysis of TUNEL foci in kidneys across five experimental groups (5 fields per mouse were analyzed, *n* = 3 mice per group). N) Quantitative assessment of CD11c^+^ immune cell infiltration in kidneys across five experimental groups (5 fields per mouse were analyzed, *n* = 3 mice per group). O) Integrated analysis of brain tissues and cognitive function across five experimental groups: H&E staining, 53BP1 foci, TUNEL assay, CD11c^+^ immune infiltration, and MWM performance. P) Quantitative analysis of 53BP1 foci in the brain across five experimental groups (5 fields per mouse were analyzed, *n* = 3 mice per group). Q) Quantitative analysis of TUNEL foci in the brain across five experimental groups (5 fields per mouse were analyzed, *n* = 3 mice per group). R) Quantitative assessment of CD11c^+^ immune cell infiltration in the brain across five experimental groups (5 fields per mouse were analyzed, *n* = 3 mice per group). S) Escape latency during the Morris water maze test across five experimental groups over a 5‐day training period (*n* = 5 mice per group). T) Platform location latency in the Morris water maze test across five experimental groups (Each mouse was tested once from each of the four quadrants, *n* = 5 mice per group). All data are presented as mean ± SEM. Statistical differences were assessed via one‐way ANOVA with Tukey's multiple comparisons test. Escape/platform latency was analyzed using repeated‐measures ANOVA. ^*^ Indicates *p* < 0.05, ^**^ indicates *p* < 0.01. Analyses were performed using GraphPad Prism 9. For the quantitative analyses in (E), ^*^ denotes *p* < 0.05, ^**^ denotes *p* < 0.01 in the comparison of fast myosin skeletal heavy chain, while # denotes *p* < 0.05, ## denotes *p* < 0.01 in the comparison of slow myosin skeletal heavy chain.

The effects of MSNs, SMSNs, and SeMSNs on organ‐level aging were further evaluated, focusing on muscle, kidney, brain, and liver tissues, key organs often affected by age‐related pathologies such as sarcopenia, chronic kidney disease, neurodegenerative disorders, and hepatic steatosis. Our results indicated that SeMSNs exhibited significantly superior efficacy in mitigating aging‐related degeneration in these organs compared to SMSNs, while MSNs alone showed no therapeutic effects. In skeletal muscle, SeMSNs treatment preserved myofiber architecture (**Figure** **3**A) and significantly reduced 53BP1^+^ DNA damage foci (*p* < 0.01 vs aged controls; Figure 3D), preventing age‐related atrophy of fast‐twitch fibers (Type II) and maintaining normal fast/slow fiber ratios (Figure 3A–E). These structural improvements correlated with preserved physical function, including enhanced grip strength (*p* < 0.01; Figure 3B) and exercise endurance (running time: *p* < 0.01; Figure 3C), alongside reduced apoptosis (TUNEL) and inflammation (CD11c^+^) to youthful levels (Figure 3A,F,G). In the kidney, SeMSNs prevented glomerular degeneration (Figure 3H), stabilized renal function (urine output, creatinine clearance and blood urea nitrogen: both *p* < 0.01; Figure 3I–K), and reduced aging‐related markers such as 53BP1^+^ DNA damage, TUNEL^+^ apoptosis, and CD11c^+^ inflammation (all *p* < 0.01; Figure 3H,L–N) to levels comparable to young controls. In the brain, SeMSNs preserved hippocampal neuronal structure (Figure 3O) and significantly reduced DNA damage (53BP1: *p* < 0.01), apoptosis (TUNEL: *p* < 0.01), and neuroinflammation (CD11c^+^: *p* < 0.01; Figure 3O,P–R) to youthful levels. Cognitive testing showed that SeMSNs‐treated mice performed similarly to young controls, exhibiting improved escape latencies and platform localization accuracy in Morris Water Maze (MWM) testing (Figure 3O,S,T) and significantly outperforming other aged groups. Immunofluorescence analysis of hepatic tissue revealed that SeMSNs effectively suppressed DNA damage, apoptosis, and inflammatory responses (Figure S4, Supporting Information). Additionally, liver staining demonstrated that SeMSNs significantly inhibited lipid accumulation in the liver (Figure S4, Supporting Information). Hematoxylin and Eosin (H&E) staining of other tissues, including the heart, liver, spleen, and lungs, showed no toxic damage from SeMSNs treatment (Figure S5, Supporting Information). We also compared the anti‐aging effects of SeMSNs and SeMet on the aforementioned organs. The results demonstrated that SeMSNs were significantly superior to SeMet in improving grip strength (Figure S6A, Supporting Information; *p* < 0.01), exercise endurance (Figure S6B, Supporting Information; *p* < 0.01), urine output (Figure S6C, Supporting Information; *p* < 0.01), and creatinine clearance rate (Figure S6D, Supporting Information; *p* < 0.01), levels in aged mice, as well as in reducing blood urea nitrogen (Figure S6E, Supporting Information; *p* < 0.01) and escape latencies and enhancing platform localization accuracy in the MWM (Figure S6F–H, Supporting Information; *p* < 0.01). These results collectively highlight the significant role of SeMSNs in resisting aging across multiple organs.

### 2.4 SeMSNs Modulate Multi‐Organ Aging Progression By Suppressing Sik1

**Figure 4:**
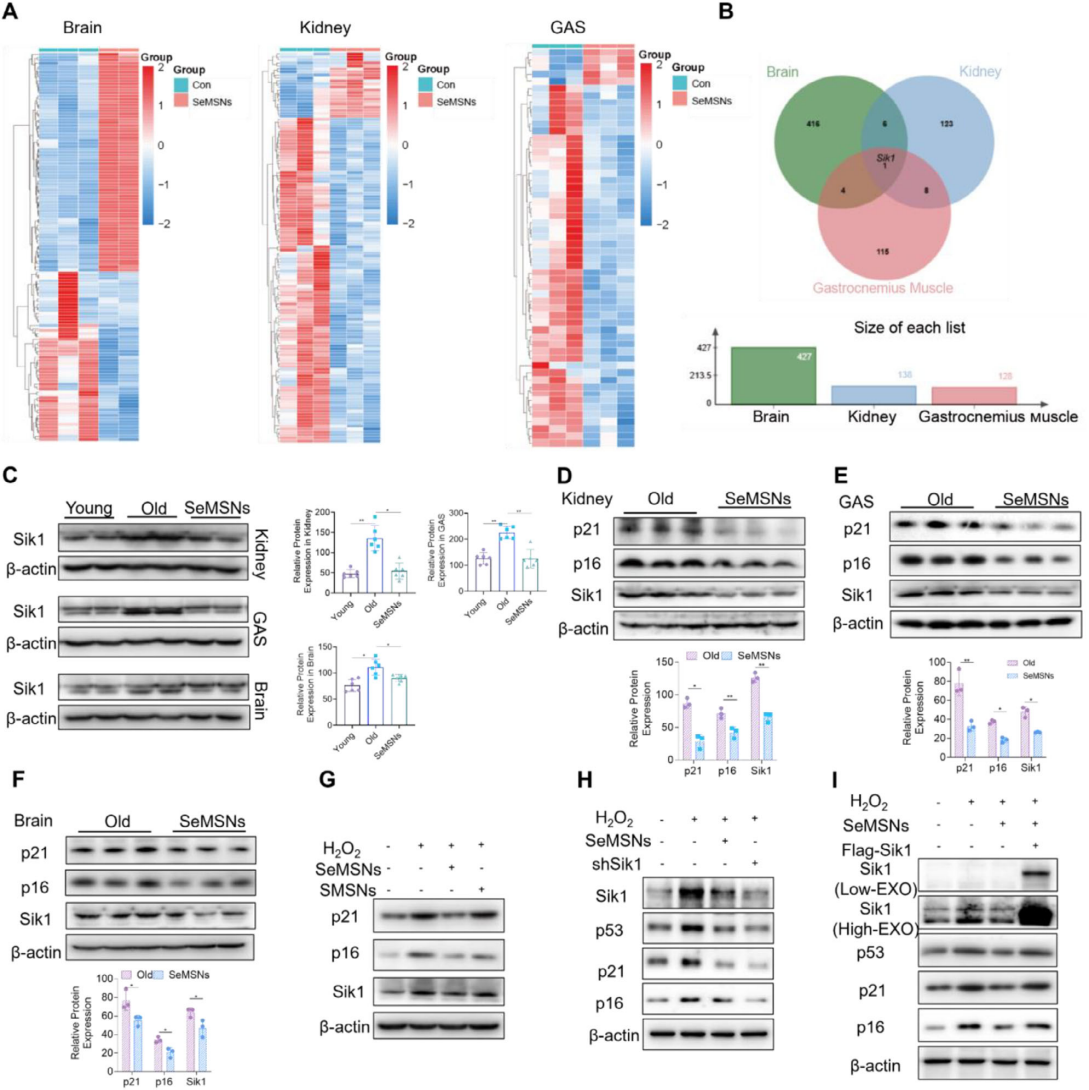
SeMSNs modulate multi‐organ aging progression by suppressing Sik1. A) Transcriptomic heatmaps of brain, kidney, and gastrocnemius muscle tissues comparing Old (*n* = 3 per tissue) and SeMSNs groups (brain: *n* = 2; kidney/muscle: *n* = 3). Color scales reflect Z‐score‐normalized gene expression. B) Venn diagram analysis of differentially expressed genes (DEGs) in brain, kidney, and gastrocnemius muscle tissues from aged mice, identifying Sik1 as the only consistently downregulated gene across all three tissues. C) Western blot analysis of Sik1 protein expression in brain, kidney, and gastrocnemius muscle tissues from Young, Old, and SeMSNs mice, normalized to β‐actin (*n* = 3 mice per group, per mice analyzed in duplicate). D) Western blot analysis of p21, p16, and Sik1 expression in brain tissue from Old and SeMSNs mice, normalized to β‐actin (*n* = 3 mice per group). E) Western blot analysis of p21, p16, and Sik1 expression in kidney tissue from Old and SeMSNs mice, normalized to β‐actin (*n* = 3 mice per group). F) Western blot analysis of p21, p16, and Sik1 expression in gastrocnemius muscle tissue from Old and SeMSNs mice, normalized to β‐actin (*n* = 3 mice per group). G) Western blot analysis of p21, p16, and Sik1 expression in HSF cells under four experimental conditions: untreated control, H_2_O_2_‐induced oxidative stress, H_2_O_2_ + SeMSNs, and H_2_O_2_ + SMSNs. Protein levels were normalized to β‐actin (*n* = 3 independent experiments). H) Western blot analysis of p53, p21, p16, and Sik1 expression in HSF cells under four experimental conditions: untreated control, H_2_O_2_‐induced oxidative stress, H_2_O_2_ + SeMSNs, and H_2_O_2_ + shSik1 (Sik1 knockdown). Protein levels were normalized to β‐actin (*n* = 3 independent experiments). I) Western blot analysis of p53, p21, p16, and Sik1 expression in HSF cells under four experimental conditions: untreated control, H_2_O_2_‐induced oxidative stress, H_2_O_2_ + SeMSNs, and H_2_O_2_ + SeMSNs with Sik1 overexpression. Protein levels were normalized to β‐actin (*n* = 3 independent experiments). All data are presented as mean ± SD. Intergroup comparisons were performed using one‐way ANOVA with Tukey's post hoc test. ^*^ *p* < 0.05, ^**^ *p* < 0.01. Analyses were performed using GraphPad Prism 9.

To investigate the conserved pathways through which SeMSNs counteract multi‐organ aging, RNA‐seq was performed on brain, kidney, and skeletal muscle tissues, followed by integration of differentially expressed genes (DEGs) to identify key molecules mediating these effects (**Figure** **4**A). Transcriptomic analysis identified Salt‐inducible kinase 1 (Sik1) as the sole DEG consistently regulated by SeMSNs across brain, kidney, and skeletal muscle tissues in aged mice (|Log_2_FC| > 1 and FDR < 0.05; Figure 4B). Western blot validation confirmed increased Sik1 protein expression in aged brain (1.5‐fold, *p* < 0.05), kidney (2.8‐fold, *p* < 0.01), and skeletal muscle (1.8‐fold, *p* < 0.01), which was reduced by SeMSNs treatment (Figure 4C). Previous studies have shown that Sik1 promotes cellular senescence by upregulating or stabilizing the expression of senescence markers p21 and p16.^[^ ^27^, ^28^ ^]^ Our results further corroborated this, with p21 and p16 expression patterns in brain, kidney, and skeletal muscle tissues and multiple cells mirroring those of Sik1, all of which were significantly suppressed by SeMSNs treatment (Figure 4D–F; Figure S7, Supporting Information). In H_2_O_2_‐treated human skin fibroblasts (HSFs), Sik1 expression was specifically downregulated by SeMSNs, but not by SMSNs (Figure 4G). To assess whether SeMSNs exert anti‐senescence effects via Sik1 modulation, *Sik1* knockout and overexpression experiments were performed. *Sik1* knockout significantly reduced p16 and p21 expression, while Sik1 overexpression completely abolished SeMSNs' inhibitory effects on H_2_O_2_‐induced p16/p21 upregulation (Figure 4H,I). Quantitative real‐time PCR (qPCR) analysis of HSFs and murine tissue samples further validated the suppressive effects of SeMSNs on *CDKN1A* (human) /*Cdkn1a* (mouse), *CDKN2A*/*Cdkn2a*, and *Sik1* transcription (Figures S8 and S9, Supporting Information). These findings establish Sik1 as a key molecular mediator through which SeMSNs exert anti‐senescence activity.

### 2.5 SeMSNs Suppress NFATc2‐Driven Sik1 Expression Through Maintaining Calcium Homeostasis in Aged Mice

**Figure 5:**
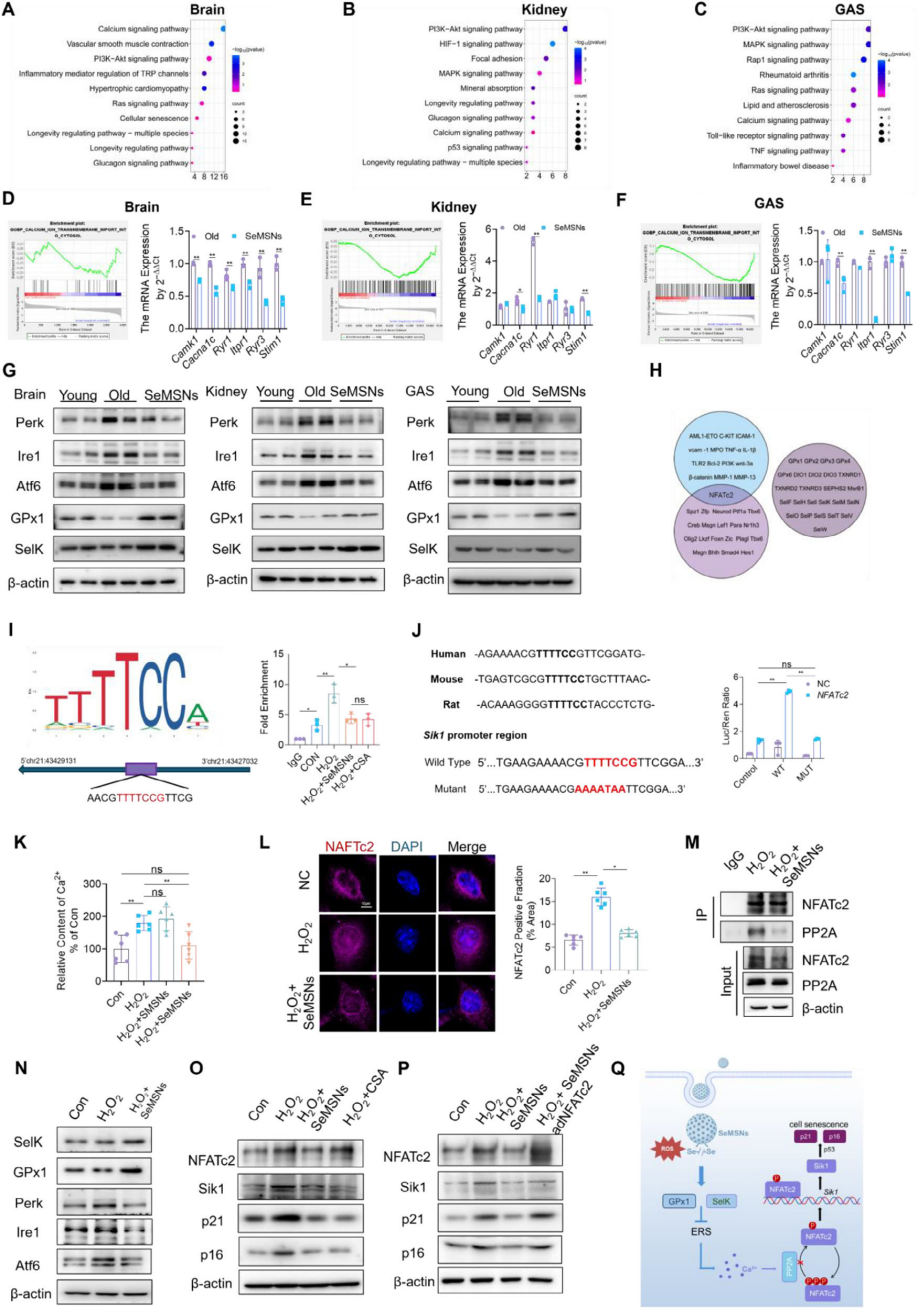
SeMSNs preserve ER proteostasis and Ca^2+^ homeostasis via selenium‐dependent upregulation of GPx1/SelK, leading to inhibition of NFATc2‐driven Sik1 gene expression in aged mice. A) KEGG pathway enrichment analysis of brain tissue‐specific differentially expressed genes (DEGs) in SeMSNs‐treated versus Old mice. Top enriched pathways were identified using Fisher's exact test with Benjamini–Hochberg correction (FDR < 0.05; brain: *n* = 2; kidney/muscle: *n* = 3). B) KEGG pathway enrichment analysis of kidney tissue‐specific DEGs in SeMSNs‐treated versus Old mice. Top enriched pathways were identified using Fisher's exact test with Benjamini–Hochberg correction (FDR < 0.05; brain: *n* = 2; kidney/muscle: *n* = 3). C) KEGG pathway enrichment analysis of gastrocnemius muscle tissue‐specific differentially expressed genes (DEGs) in SeMSNs‐treated versus Old mice. Top enriched pathways were identified using Fisher's exact test with Benjamini–Hochberg correction (FDR < 0.05; brain: *n* = 2; kidney/muscle: *n* = 3). D) GSEA and qPCR analysis of calcium signaling genes in SeMSNs‐treated versus Old brains (brain: *n* = 2; kidney/muscle: *n* = 3). E) GSEA and qPCR analysis of calcium signaling genes in SeMSNs‐treated versus Old kidneys (brain: *n* = 2; kidney/muscle: *n* = 3). F) GSEA and qPCR analysis of calcium signaling genes in SeMSNs‐treated versus Old gastrocnemius muscle (brain: *n* = 2; kidney/muscle: *n* = 3). G) Western blot analysis of ER stress markers (Perk, Ire1, Atf6) and selenoproteins (GPx1, SelK) in brain, kidney, and gastrocnemius muscle tissues from Young, Old, and SeMSNs‐treated mice. Protein levels normalized to β‐actin (*n* = 3 mice per group, per mice analyzed in duplicate). H) Venn diagram analysis integrating three gene subsets: selenoproteins, selenium‐associated proteins, and transcriptional regulators of Sik1. I) JASPAR‐predicted NFATc2 binding to the Sik1 promoter validated by ChIP‐qPCR (*n* = 3 samples per group). J) Sequence alignment of predicted transcription factor binding sites (NFATc2) in Sik1 promoters from human, mouse, and rat. Mutated nucleotides (red) disrupt core motifs. Dual‐luciferase reporter assays comparing wild‐type (WT) and mutant (Mut) Sik1 promoter activity (*n* = 3 samples per group). K) Quantification of intracellular calcium ion levels in HSF cells under four experimental conditions: Control, H_2_O_2_, H_2_O_2_ + SMSNs, and H_2_O_2_ + SeMSNs (*n* = 6 samples per group). L) NFATc2 subcellular localization and quantitative analysis in cells under three experimental conditions: NC, H_2_O_2_, and H_2_O_2_ + SeMSNs (2 fields per sample were analyzed, *n* = 3 sample per group). M) Co‐IP analysis showing H_2_O_2_ enhances PP2A‐NFATc2 binding, reversed by SeMSNs (*n* = 3 independent experiments). N) Western blot analysis of ER stress markers (Perk, Ire1, Atf6) and selenoproteins (GPx1, SelK) in HSF cells under three experimental conditions: Con, H_2_O_2_, and H_2_O_2_ + SeMSNs (*n* = 3 independent experiments). O) Western blot analysis of NFATc2, Sik1, p21, and p16 in HSF cells under four experimental conditions: Con, H_2_O_2_‐induced oxidative stress, H_2_O_2_ + SeMSNs, and H_2_O_2_ + CSA. Protein levels normalized to β‐actin (*n* = 3 independent experiments). P) Western blot analysis of NFATc2, Sik1, p21, and p16 in HSF cells under four experimental conditions: Con, H_2_O_2_‐induced oxidative stress, H_2_O_2_ + SeMSNs, and H_2_O_2_ + SeMSNs + adNFATc2. Protein levels normalized to β‐actin (*n* = 3 independent experiments). Q) Diagram illustrating the SeMSNs anti‐aging mechanism. All data are presented as mean ± SD. Statistical differences were assessed via one‐way ANOVA with Tukey's multiple comparisons test. ^*^ *p* < 0.05, ^**^ *p* < 0.01. Analyses were performed using GraphPad Prism 9.

To elucidate the mechanism by which SeMSNs inhibit Sik1 expression in aging organs, pathway enrichment analysis was performed on DEGs from brain, kidney, and skeletal muscle tissues in both aged and SeMSNs‐treated groups. Notably, the calcium signaling pathway was consistently identified among the top 10 enriched pathways across all three tissues (**Figure** **5**A–C). Further analysis through gene set enrichment analysis (GSEA) and qPCR revealed synergistic suppression of critical calcium homeostasis regulators following SeMSNs treatment (Figure 5D–F). The endoplasmic reticulum (ER) serves as the primary intracellular calcium reservoir, and its calcium homeostasis is tightly regulated by the unfolded protein response (UPR).^[^ ^29^ ^]^ Analysis of key UPR components, Protein kinase RNA‐like ER kinase (Perk), Inositol‐requiring enzyme 1 (Ire1), and Activating transcription factor 6 (Atf6), revealed significant activation of UPR signaling in aged brain, kidney, and skeletal muscle tissues. SeMSNs treatment effectively suppressed UPR hyperactivation in these aged tissues, restoring UPR activity to levels comparable to those in normal adult samples (Figure 5G). Among the 25 known selenoproteins, glutathione peroxidase 1 (GPx1) and selenoprotein K (SelK) mitigate oxidative stress‐induced UPR activation by scavenging ROS in the ER lumen.^[^ ^30^, ^31^ ^]^ This study demonstrated significant downregulation of GPx1 and SelK in aged brain, kidney, and skeletal muscle tissues, whereas SeMSNs supplementation effectively restored their expression levels (Figure 5G). Additionally, compared to SeMet, SeMSNs exhibited a longer duration of activation toward both GPx1 and SelK, along with lower cytotoxicity (Figure S10, Supporting Information).

To determine whether SeMSNs modulate Sik1 expression by maintaining calcium homeostasis, a systematic investigation was conducted. SeMSNs significantly inhibited Sik1 expression at the transcriptional level (Figures S8 and S9, Supporting Information). Subsequently, potential *Sik1* transcription factors were predicted using the JASPAR database (with a stringent cutoff score > 8), followed by integrative analysis with selenoprotein groups and calcium signaling pathway genes. Notably, the transcription factor nuclear factor of activated T cells 2 (NFATc2) was identified as a common calcium ion‐responsive regulator in both the core calcium signaling pathway and the predicted Sik1 transcription factors. These results suggest that SeMSNs may indirectly influence Sik1 expression via the calcium signaling‐NFATc2 axis rather than directly regulating Sik1 through selenoproteins (Figure 5H). The study further demonstrates that NFATc2 directly regulates the transcriptional expression of the Sik1 gene. Chromatin immunoprecipitation (ChIP) assays confirmed that NFATc2 specifically binds to the Sik1 promoter region (43 427 031 to 43 429 131 bp). Dual‐luciferase reporter assays revealed that *NFATc2* overexpression significantly enhanced Sik1 promoter activity (≈5.8‐fold, *p* < 0.01), while mutation of the NFATc2 binding site completely abolished this activation (Figure 5I,J). These findings not only elucidate NFATc2's role as a transcription factor for Sik1 but also demonstrate its function as a positive regulator of Sik1 expression. In HSFs, SeMSNs maintained intracellular calcium concentrations at physiological levels (no significant difference vs control, *p* > 0.05), while SMSNs had no such effect (Figure 5K). SeMSNs treatment inhibited NFATc2 nuclear translocation, with immunofluorescence showing that H_2_O_2_ stimulation increased NFATc2 nuclear localization by 2.3‐fold (*p* < 0.01), whereas SeMSNs treatment resulted in only a 1.2‐fold increase (*p* < 0.05 vs control; Figure 5L). Co‐immunoprecipitation assays confirmed that SeMSNs reduced the interaction between NFATc2 and protein phosphatase 2A (PP2A), thereby decreasing NFATc2 dephosphorylation (Figure 5M). SeMSNs also significantly upregulated antioxidant selenoproteins and restored ER stress markers to near‐normal levels (Figure 5N). Rescue experiments further confirmed that SeMSNs inhibit cellular senescence by regulating Sik1 through the maintenance of intracellular calcium homeostasis. Treatment with the calcineurin inhibitor cyclosporine A (CSA) significantly ameliorated the aberrant expression of Sik1, p21, and p16 in H_2_O_2_‐stimulated HSF cells (Figure 5O). The anti‐senescence protective effects of SeMSNs were significantly counteracted when co‐administered with *NFATc2* overexpression (Figure 5P). These results validate the critical role of the “SeMSNs‐calcium homeostasis‐NFATc2‐Sik1” axis in regulating cellular senescence (Figure 5Q).

### 2.6 Selenium Supplementation Mediates Sik1 Involvement in Anti‐Aging Regulation with Clinical Relevance

**Figure 6:**
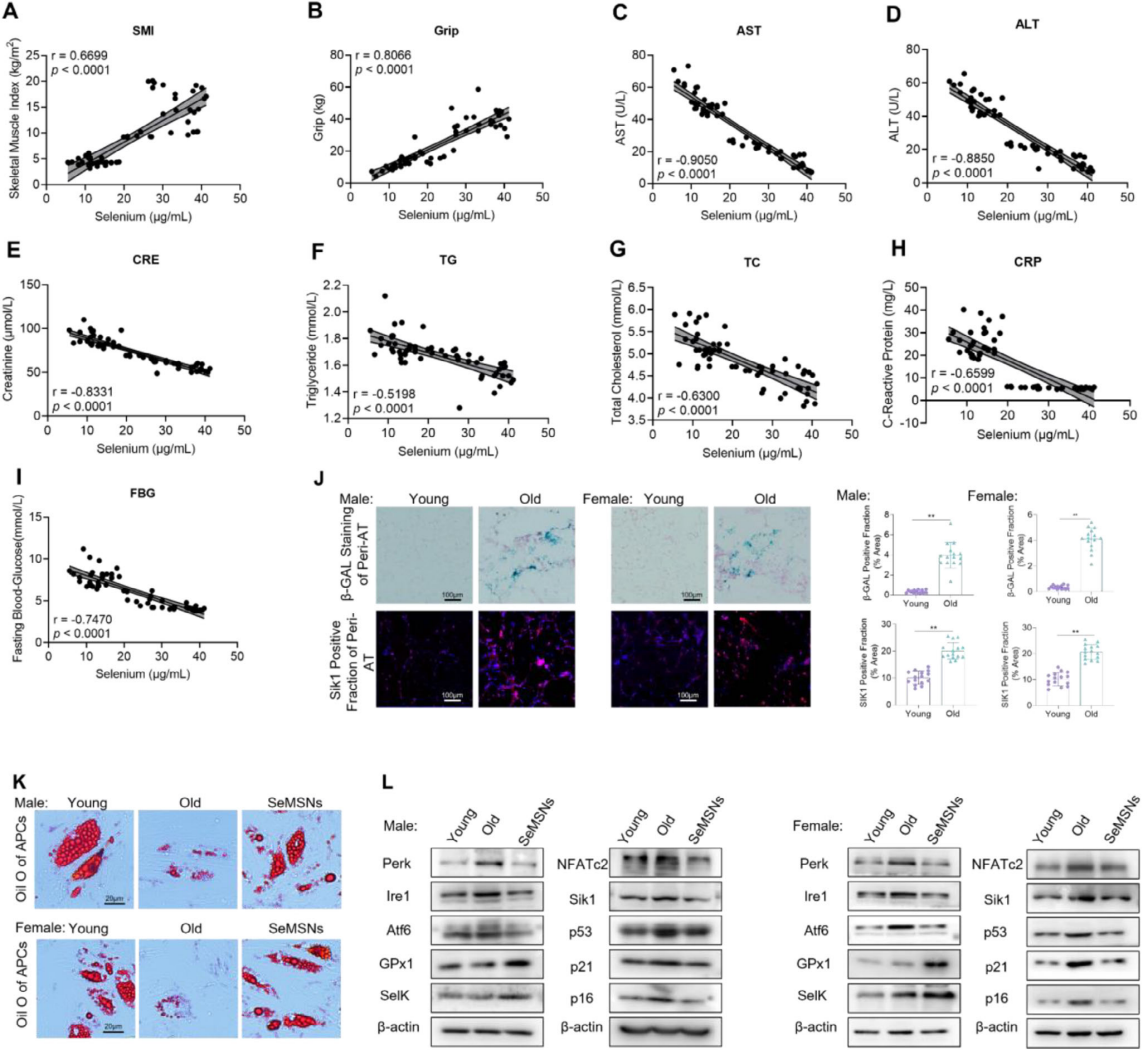
Circulating selenium correlates with aging progression, and SeMSNs enhance the functionality of aged human subcutaneous adipose progenitors. A) Correlation between serum selenium levels and skeletal muscle index in the young group (*n* = 30) versus the old group (*n* = 30). B) Correlation between serum selenium levels and grip strength in the young group (*n* = 30) versus the old group (*n* = 30). C) Correlation between serum selenium levels and AST in the young group (*n* = 30) versus the old group (*n* = 30). D) Correlation between serum selenium levels and ALT in the young group (*n* = 30) versus the old group (*n* = 30). E) Correlation between serum selenium levels and creatinine in the young group (*n* = 30) versus the old group (*n* = 30). F) Correlation between serum selenium levels and triglyceride levels in the young group (*n* = 30) versus the old group (*n* = 30). G) Correlation between serum selenium levels and total cholesterol in the young group (*n* = 30) versus the old group (*n* = 30). H) Correlation between serum selenium levels and C‐reactive protein in the young group (*n* = 30) versus the old group (*n* = 30). I) Correlation between serum selenium levels and fasting blood glucose in the young group (*n* = 30) versus the old group (*n* = 30). J) β‐GAL staining in peri‐adipose tissue (Peri‐AT) from young and old male and female adults. Sik1 immunofluorescence in Peri‐AT sections from young and old male and female adults. Each group consists of five samples, and for each sample, three fields of view are selected. K) Oil Red O staining of lipid droplets in primary adipocytes from the Young, Old, and SeMSNs old groups. L) Western blot analysis of ER stress markers (Perk, Ire1, Atf6), selenoproteins (GPx1, SelK), calcium signaling components (NFATc2), Sik1, and senescence‐associated proteins (p53, p21, p16) in three experimental groups: Young, Old, and SeMSNs. Protein levels normalized to β‐actin. All data are presented as mean ± SEM. Correlation analysis was performed using simple linear regression. Statistical differences were assessed via one‐way ANOVA with Tukey's multiple comparisons test. ^*^ *p* < 0.05, ^**^ *p* < 0.01. Analyses were performed using GraphPad Prism 9.

Building upon our findings regarding SeMSNs' anti‐aging effects at both cellular and animal levels, this study further evaluated: 1) the correlation between blood selenium levels and human aging in population studies, and 2) the clinical potential of SeMSNs in combating human tissue aging using isolated human APCs. In the clinical correlation analysis of blood selenium levels, the study enrolled 30 healthy elderly participants and 30 age‐matched healthy young controls. Clinical data revealed significantly lower serum selenium levels in elderly participants compared to young controls (*p* < 0.01; Table S1, Supporting Information). Serum selenium concentrations showed strong positive correlations with both skeletal muscle index (*r* = 0.6699, *p* < 0.0001; **Figure** **6**A) and grip strength (*r* = 0.8066, *p* < 0.0001; Figure 6B), suggesting selenium's role in maintaining muscle function during aging. Additionally, serum selenium levels exhibited significant inverse correlations with hepatic function markers (AST: *r* = −0.9050, *p* < 0.0001; ALT: r = −0.8850, *p* < 0.0001), indicating that selenium may exert anti‐aging effects on the liver through attenuation of hepatocyte damage (Figure 6C,D). Furthermore, a significant inverse correlation was found between serum selenium levels and creatinine (*r* = −0.8331, *p* < 0.0001; Figure 6E), suggesting a renoprotective role for selenium in aging. Serum selenium levels also demonstrated significant inverse correlations with multiple metabolic and inflammatory markers, including triglycerides (TG: *r* = −0.5198, *p* < 0.0001), total cholesterol (TC: *r* = −0.6300, *p* < 0.0001), C‐reactive protein (CRP: *r* = −0.6599, *p* < 0.0001), and FBG (*r* = −0.7470, *p* < 0.0001). These results suggest that selenium may exert broad anti‐aging effects through concurrent modulation of metabolic homeostasis and inflammatory responses (Figure 6F–I).

The adipose tissue staining results revealed that the expression of β‐galactosidase (male: 12.1‐fold; female: 12.0‐fold; *p* < 0.01) and Sik1 (male: 2.6‐fold; female: 2.0‐fold; *p* < 0.01) in subcutaneous adipose tissue was significantly higher in elderly patients compared with younger patients (Figure 6J). Subcutaneous adipose‐derived APCs isolated from elderly male and female patients exhibited markedly reduced adipogenic differentiation capacity. However, treatment with SeMSNs restored their adipogenic potential (Figure 6K). Additionally, SeMSNs treatment markedly upregulated the expression of GPx1 and SelK in aged APCs and effectively restored levels of ER stress markers (Perk, Ire1, Atf6) and calcium signaling proteins (NFATc2) to physiological levels. Importantly, SeMSNs treatment significantly downregulated the senescence‐associated factor Sik1 and cellular aging markers (p53, p21, p16), suggesting a pleiotropic anti‐aging mechanism (Figure 6L). The regulatory role of this signaling pathway is significant for APC cells derived from both sexes. These findings establish the anti‐aging properties of clinical selenium and mechanistically link the “SeMSNs‐calcium‐NFATc2‐Sik1” pathway to human adipose tissue aging.

## 3 Discussion

Aging is a significant risk factor for age‐related diseases, highlighting the importance of developing nutritional supplements targeting aging mechanisms to extend lifespan and improve health in the elderly. Studies show that blood selenium levels in individuals over 60 years old are significantly lower than in younger adults, with a more pronounced decline after age 70.^[^ ^32^ ^]^ Our previous work demonstrated that self‐synthesized SeMSNs are an effective sustained‐release selenium supplement, offering superior safety and efficacy in treating sarcopenia compared to commercially available SeMet at equivalent doses. In the present study, SeMSNs were further applied to anti‐aging research, revealing that: 1) SeMSNs significantly extended lifespan and improved aging‐associated functional decline in the brain, skeletal muscle, kidney, liver, adipose tissue, and skin; 2) SeMSNs restored intracellular Ca^2+^ homeostasis across multiple aged tissues; 3) SeMSNs upregulated selenoproteins (SelK and GPx1), mitigating ROS accumulation and ER stress‐induced Ca^2+^ release. This process suppressed NFATc2‐mediated Sik1 transcription, thereby reducing the accumulation of senescence markers p16 and p21.

Numerous clinical studies have established an association between selenium and longevity, though this relationship is not linear. Selenium deficiency is strongly linked to various aging‐related diseases, including neurodegenerative disorders, cardiovascular diseases, immune dysfunction, and skin aging.^[^ ^33^ ^]^ Epidemiological data show an inverse correlation between blood selenium levels and ischemic heart disease mortality.^[^ ^34^ ^]^ A study in Maryland found a significant association between serum selenium levels and grip strength in elderly women, while low serum selenium and carotenoid levels were shown to increase mortality risk among older women.^[^ ^23^, ^35^, ^36^ ^]^ Our clinical samples confirmed the inverse correlation between serum selenium levels and aging. However, due to selenium's narrow therapeutic window, this relationship is non‐linear.^[^ ^37^ ^]^ Our previous study demonstrated that, at equivalent doses, SeMSNs offer more stable selenium supplementation, superior safety, and enhanced therapeutic efficacy compared to commercially available SeMet in the treatment of sarcopenia.^[^ ^23^ ^]^ The present work further reveals that SeMSNs exert anti‐aging effects at the cellular level, exhibiting significant senotherapeutic activity with an improved safety profile.

Excessive selenium intake can result in selenosis, manifesting as diarrhea, alopecia, chills, tremors, and, in severe cases, neurological damage, hepatic lesions, pulmonary edema, or even death.^[^ ^38^ ^]^ Nano‐selenium presents a promising alternative for anti‐aging selenium supplementation due to its enhanced bioavailability and controlled, targeted release capabilities. Dietary supplementation with nano‐selenium has been shown to increase selenium deposition in Japanese quail testes and ovaries, improving reproductive performance.^[^ ^39^ ^]^ Chitosan/citrate‐complexed nano‐selenium has also effectively protected mice against ROS damage in galactose‐induced aging models.^[^ ^40^ ^]^ However, systematic research on the effects of selenium or nano‐selenium on aging processes and multi‐organ aging remains limited. This study employed a novel nano‐selenium formulation (SeMSNs), designed to overcome the limitations of traditional antioxidant nano‐selenium by focusing on maintaining redox balance in aged organisms. The SeMSNs employed in this study demonstrate significantly enhanced colloidal stability at room temperature, effectively resisting aggregation and sedimentation. Capitalizing on the inherent antibacterial properties of chitosan (CTS), these nanoparticles exhibit reduced toxicity and improved safety profiles. Furthermore, unlike conventional CTS‐SeNPs, the present SeMSNs feature a diselenide (‐Se‐Se‐) bridged architecture that confers dual‐responsive drug release capabilities, triggered by both oxidative and reductive stimuli. Their high specific surface area and well‐defined mesoporous structure render them particularly suitable for loading macromolecular therapeutics.^[^ ^26^, ^41^ ^]^ Notably, the synthetic protocol ensures excellent batch‐to‐batch reproducibility, underscoring its robustness for scalable production. This selenium supplementation strategy effectively elevates serum selenium levels in the elderly to a range comparable to that of younger populations. Our study further demonstrated that in aged mice, administration of SeMSNs significantly increased selenium concentrations in multiple tissues, including skeletal muscle, liver, kidney, spleen, and brain, within just 2 h. These results indicate that SeMSNs not only efficiently replenish selenium reserves in visceral organs of aging subjects but also possess the ability to cross the blood‐brain barrier.

We systematically evaluated the anti‐aging effects of SeMSNs in aging mice across three dimensions: lifespan extension, mitigation of physical frailty, and multi‐organ aging regulation. The results demonstrated that SeMSNs were significantly more effective than SeMet and SMSNs. In terms of lifespan, SeMSNs markedly improved survival rates, outperforming both SMSNs and conventional MSNs. For healthspan assessment, comprehensive frailty index evaluations, including body weight, coat condition, body composition, sensory function, and digestive/urinary performance, revealed optimal improvement in the SeMSNs group. Regarding multi‐organ aging, SeMSNs substantially reduced aging markers, DNA damage, and oxidative stress in the brain, skeletal muscle, kidney, liver, adipose tissue, and skin, accompanied by enhanced organ function. These findings provide a holistic assessment of the multi‐faceted anti‐aging benefits of selenium supplementation and offer critical insights into the geroprotective potential and clinical applicability of nano‐selenium. Although SMSNs exhibited modest anti‐aging effects, likely attributable to indirect antioxidant activity through thiol group release (particularly glutathione, GSH) following disulfide bond cleavage, this regulation remained relatively weak.^[^ ^42^ ^]^ Recent evidence also indicates a role of SMSNs in maintaining proteostasis via thiol‐disulfide exchange reactions that balance MuRF1 and MAFbx ubiquitin ligase activities, thereby suppressing excessive muscle protein degradation.^[^ ^43^ ^]^ However, the antioxidant response mediated by SMSNs is slow and indirect. In contrast to SeMSNs, which exert therapeutic effects through active selenium release, SMSNs primarily function as delivery vehicles. Thus, their potential in anti‐aging applications was not further investigated in this study. Compared to commercially available SeMet, which is prone to oxidation, has a narrow therapeutic window, and distributes unevenly in vivo, SeMSNs demonstrated superior safety and pharmacological properties. Their slow degradation in vivo ensures sustained selenium release, enabling stable activation of key selenoproteins (GPx1 and SelK) and highlighting enhanced biological efficacy and translational potential.

Age serves as a common pathological foundation for numerous aging‐related diseases. However, the precise pathogenic mechanisms and shared regulatory targets remain incompletely understood. Our findings, demonstrating that SeMSNs ameliorate multi‐organ aging phenotypes, are supported by multi‐organ transcriptomic sequencing analysis, which, for the first time, highlights the systemic regulatory role of selenium supplementation in calcium homeostasis. Dysregulated calcium homeostasis has become a hallmark of aging: As a critical second messenger, Ca^2+^ is tightly regulated by the ER to maintain resting concentrations (50–100 nm).^[^ ^44^, ^45^ ^]^ However, aging leads to the functional decline of calcium regulatory systems, characterized by elevated resting Ca^2+^ levels and diminished stimulus‐evoked calcium transient amplitudes.^[^ ^46^, ^47^ ^]^ This dysregulation is closely associated with multi‐system aging‐related pathologies: increased Ca^2+^ in hippocampal and cortical neurons disrupts CaMKII/calcineurin signaling, impairing learning and memory;^[^ ^48^ ^]^ calcium overload in cardiomyocytes triggers mitochondrial permeability transition pore opening and apoptosis;^[^ ^49^, ^50^ ^]^ and in bone tissue, excess calcium upregulates osteoclast marker genes (e.g., TRAP, CTSK) via the NFATc1 signaling pathway, enhancing bone resorption.^[^ ^51^, ^52^ ^]^ Notably, our study reveals that SeMSNs regulate calcium homeostasis through a dual mechanism: First, they markedly upregulate SelK, an ER stress‐protective protein, enhancing ER calcium handling. Second, SeMSNs boost the expression of the antioxidant enzyme GPx1, preserving calcium pump activity by eliminating ROS.^[^ ^53^, ^54^ ^]^ This is critical, as GPx1 deficiency results in cytotoxic H_2_O_2_ accumulation, triggering apoptotic cell death. These findings provide novel insights into the pivotal role of the selenium‐calcium axis in aging regulation.

To identify common targets of SeMSNs in combating multi‐organ aging, the DEGs in aged tissues were intersected after SeMSNs intervention. This analysis revealed that SeMSNs consistently downregulated Sik1 across multiple aging organs. Sik1, an AMPK‐related kinase, plays a crucial role in energy metabolism, cell proliferation, and stress responses.^[^ ^55^, ^56^, ^57^ ^]^ Sik1 can directly phosphorylate p21, altering its stability or subcellular localization and promoting its nuclear retention.^[^ ^58^, ^59^ ^]^ Additionally, Sik1 inhibits the activity of CRTC2/3, suppressing CREB‐mediated pro‐proliferative gene expression and indirectly upregulating p21 through a p53‐dependent pathwa.^[^ ^60^, ^61^ ^]^ This study observed that Sik1 transcription is significantly enhanced in aged tissues, whereas SeMSNs intervention reduces Sik1 at the transcriptional level, leading to the downregulation of p16 and p21 in both aged tissues and cells.

This study further explored whether selenium‐mediated calcium ion homeostasis contributes to the transcriptional activation of Sik1, thus ameliorating multi‐organ aging phenotypes. Using the JASPAR database, potential transcription factors of Sik1 were predicted, revealing NFATc2 as a likely participant in Ca^2+^‐dependent Sik1 transcriptional activation. Previous studies have shown that NFATc2 increases with age in articular chondrocytes. During development, the absence of NFATc2 does not affect joint formation, but its overexpression in adulthood and old age results in severe dysfunction of articular chondrocytes.^[^ ^62^ ^]^ Another study reported elevated total levels and nuclear translocation of NFATc2 in both neuronal and non‐neuronal cells of patients with synucleinopathy, where its activation triggers a neuroinflammatory cascade.^[^ ^63^ ^]^ Under resting conditions, NFATc2 remains highly phosphorylated in the cytoplasm. However, upon stimulation, such as calcium influx, TNF‐α, or TGF‐β that induces calcium oscillations, NFATc2 undergoes dephosphorylation and translocates into the nucleus to activate its transcriptional activity.^[^ ^64^, ^65^ ^]^ This study revealed that in aged tissues and cellular models, NFATc2 binds more to its phosphatase PP2A and exhibits increased nuclear translocation. Additionally, NFATc2 was confirmed to act as a transcription factor for Sik1, promoting Sik1 upregulation and cellular senescence. In contrast, SeMSNs inhibit NFATc2 dephosphorylation and transcriptional activity by preserving endoplasmic reticulum homeostasis and maintaining oxidative stress balance, thus suppressing Sik1‐mediated cell cycle arrest and delaying cellular senescence.

## 4 Conclusion

In conclusion, SeMSNs are a highly effective and safe selenium supplement with multi‐organ anti‐aging benefits. By restoring intracellular calcium homeostasis, enhancing selenoprotein (SelK and GPx1) expression, and reducing oxidative stress and endoplasmic reticulum dysfunction, SeMSNs mitigate NFATc2‐mediated Sik1 transcriptional activation, alleviating cellular senescence markers (p16/p21) and improving tissue function. Notably, SeMSNs extend lifespan and enhance healthspan in aged mice, outperforming conventional antioxidant‐based selenium formulations. These findings underscore the critical role of selenium‐calcium crosstalk in aging regulation and position SeMSNs as a promising geroprotective intervention. This study has several limitations that should be considered. The intervention in aging mice was initiated at 18 months of age and maintained for 5 months, which aligns with the WHO recommendation that selenium supplementation is most beneficial from ≈50 years of age in humans. However, the human participants recruited in this study were all over 65 years old, creating a non‐negligible age gap between the animal model and the clinical cohort. Although no significant toxicity was detected in major organs after 5 months of SeMSNs treatment, more systematic assessments of acute and chronic toxicity will be essential in future translational studies. Furthermore, only male mice were used in the animal experiments to avoid potential confounding effects from the estrous cycle in females, a 4–5 day cycle during which fluctuations in estrogen and progesterone are known to directly influence key aging‐related biomarkers. In contrast, the human study included both males and females. Although SeMSNs enhanced the differentiation potential of aged adipose progenitor cells in all subjects, more data are needed to support sex‐specific comparisons. It is also worth noting that while selenium‐based nanomaterials like SeMSNs hold promise for clinical translation, existing studies have focused primarily on patients with advanced cancer. Substantial additional safety evidence will be required to support their application as nutritional supplements in the general aged population. Nevertheless, our results suggest that nano‐selenium formulations such as SeMSNs, with their controlled release properties and superior bioavailability, may overcome the limitations of traditional selenium supplements, offering a safer and more effective strategy for aging intervention.

## 5 Experimental Section

### Patient Samples

This study enrolled 30 elderly participants (≥ 65 years) and 30 young controls (18–35 years). Inclusion criteria for both groups included good general health, absence of major chronic diseases, BMI between 18.5 and 30 kg m^−2^, and the capacity to provide informed consent. Exclusion criteria were: 1) major surgery or trauma within the past 6 months; 2) use of immunosuppressants, hormones, or metabolism‐affecting drugs; 3) coagulation disorders or anticoagulant use; 4) psychiatric or cognitive impairment; 5) pregnancy or lactation; 6) participation in other trials within the last 3 months. Additional exclusions for the elderly group included severe osteoporosis, a history of fractures, advanced arthritis, or mobility impairment. Plasma samples were collected from geriatric inpatients at China Medical University‐affiliated hospitals. This part of the study was approved by the Ethics Committee of the First Affiliated Hospital of China Medical University (Shenyang, China) (project identification code: AF‐SOP‐07‐1.0‐01). Peri‐adipose tissue samples were obtained from 6 elderly and 6 young surgical patients, with ethical approval granted by the Ethics Committee of China Medical University (Shenyang, China) (Ethics approval: [2025] No.15). All participants provided written informed consent before the initiation of the study.

### Construction of Aging Mouse Model

Wild‐type male C57BL/6 mice, aged 5 and 18 months, were used in this study. The experiment included 3 experimental groups and 2 control groups, with 6–10 mice per group. Control mice received a normal diet, while experimental groups were treated with MSN, SMSNs, or SeMSNs at a concentration of 1 mg mL^−1^ and a dose of 10 mg kg^−1^ body weight, administered every two days. After the mice entered the aging period (21 months), grip strength and endurance were evaluated using a grip strength tester and a running wheel, respectively. Additional assessments included body weight, body composition, glucose tolerance, learning/memory ability, and tissue/organ damage. After all parameters had been retested at 23 months of age, the mice were humanely euthanized, and gastrocnemius muscles were weighed. Blood serum, urine, and major organs (brain, heart, liver, spleen, lung, and kidney) were collected for safety evaluation. Parallel subgroups (*n* = 10/group) were set up for continuous intervention and recording of natural survival endpoints. In addition, four groups of mice (Young, Old, SeMSNs, SeMet, *n* = 6 mice per group) were established to compare the anti‐aging effects of SeMSNs and SeMet (4.2 mg kg^−1^ day^−1^ administered via gavage, containing an equivalent selenium dosage as 10 mg kg^−1^ SeMSNs). Each group included six mice. After 5 months of treatment, assessments including grip strength, endurance capacity, water maze performance, as well as measurements of urine volume, creatinine clearance rate and serum urea nitrogen levels were carried out. Survival differences among the groups were analyzed using Kaplan–Meier survival curves, with significance evaluated by the Log‐rank test. All C57BL/6J mice used in this study were obtained from Beijing HFK Bioscience Co., Ltd., Beijing, China (Certificate No.: SCXK(Jing)2014‐0004). All animal experiments were approved by the Institutional Animal Care and Use Committee at China Medical University (Approval No.: CMU2021607).

### Construction of Cell Senescence Models

MEFs were isolated from pregnant mice (13–14 days of gestation) through humane euthanasia, followed by disinfection in 75% alcohol. The uterus was then extracted and transferred to a sterile workbench. Embryonic heads, limbs, and internal organs were removed, and the remaining trunk portions were washed with PBS before being placed in a new centrifuge tube. Trypsin was added to dissociate the tissue into single cells, which were then suspended in DMEM medium containing 15% fetal bovine serum (FBS) to halt digestion. After centrifugation, the supernatant was discarded, and the pellet was resuspended in fresh medium and evenly distributed into Petri dishes. Simultaneous treatment during passage: Cells were continuously exposed to 10 µg mL^−1^ of drug during passaging. Post‐passage treatment: Following passaging, cells were treated with 10 µg mL^−1^ of drug for 24 h. HSFs underwent replicative senescence via natural passaging. Additionally, oxidative stress‐induced senescence models were generated by treating HEK‐293T cells, HSF cells, HT‐22 cells, skeletal muscle C2C12 myoblasts, RAW264.7 cells and HCAECs with 100 mm H_2_O_2_ for 2 h, followed by 24 h of recovery in complete culture medium. Extraction of APCs: Subcutaneous adipose tissue was aseptically harvested, washed repeatedly with pre‐chilled PBS containing 2% penicillin‐streptomycin to remove erythrocytes and impurities, and minced. Enzymatic digestion was initiated by adding 0.2% (w/v) type I collagenase. After digestion, the mixture was centrifuged to pellet the cells, with the lipid layer and supernatant carefully discarded. The pellet was resuspended in F12 medium supplemented with 15% FBS, followed by centrifugation. The final pellet was resuspended in complete growth medium. Oxidative stress‐induced senescence models were also established by treating APCs with 100 mm H_2_O_2_ for 2 h, followed by a 24 h recovery period in complete culture medium.

### Optimized Synthesis Protocol for MSNs, SMSNs, and SeMSNs

Three types of materials were synthesized following previous reports.^[^ ^26^ ^]^ MSNs were prepared by dissolving hexadecyltrimethylammonium chloride (CTAC, 2 g) and triethanolamine in deionized water (20 mL) under vigorous stirring at 95 °C for 20 min. Then, silicic acid (1 g) and bis[3‐(triethoxysilyl) propyl] tetrasulfide (1.3 g) were sequentially added, with continued stirring at 95 °C for 4 h. The product was centrifuged, washed with ethanol/water, and treated twice with 10% (v/v) HCl‐ethanol solution (78 °C, 12 h per cycle) to remove surfactants before vacuum drying. For SMSNs and SeMSNs, CTAC (0.6 g) and triethanolamine (0.15 g) were dissolved in deionized water (40 mL) at 80 °C for 30 min, followed by dropwise addition of tetraethyl orthosilicate (TEOS, 4.0 g) and either bis[3‐(triethoxysilyl) propyl] tetrasulfide (1.0 g) or bis[3‐(triethoxysilyl) propyl]diselenide (1.0 g). After stirring at 80 °C (1000 rpm, 4 h), the nanoparticles were centrifuged, washed with ethanol, and subjected to template removal by refluxing in 1% NH_4_NO_3_‐ethanol solution (12 h) prior to vacuum drying. The morphology and H_2_O_2_‐triggered degradation (100 µm, 3 days) were analyzed using transmission electron microscopy (TEM). Elemental composition was determined by inductively coupled plasma mass spectrometry (ICP‐MS, Agilent), and nanoparticle size and zeta potential were assessed via dynamic light scattering (Zetasizer, Malvern) to confirm colloidal stability.

### Detection of Selenium Accumulation and Release Quantity

The experiment included a control group (basic SIF, pH 6.8) and an experimental group (SIF + H_2_O_2_). Each group consisted of three parallel samples, with 10 mg of SeMSNs added to each. The samples were incubated at 37 °C under constant temperature and dark conditions with shaking at 100 rpm. Aliquots (1 mL) were collected at time points (0, 3, 6, 12, 24, 36, and 48 h), followed by centrifugation at 12 000 rpm. The supernatant was collected, and equal volumes of fresh medium were added to maintain the dynamic release system. Selenium content in the supernatant was quantified using ICP‐MS. Cumulative release rates were calculated and compared between groups to assess differences in release kinetics. The impact of an oxidative stress environment on the sustained‐release performance of the drug was also analyzed.

### Frailty Assessment

After one month of intervention, two researchers conducted a blinded assessment of the frailty index. The assessment included the following criteria: hair condition (luster, depilation area, scored 0–2 points), body morphology (weight change, muscle atrophy, and degree of hunchback), eye‐nose condition (discharge, conjunctival congestion), digestive tract function (fecal consistency, perianal contamination), and urinary reproductive tract condition (discharge, swelling, or abnormal urination). Each item was scored on a scale of 0–2 points. Data were recorded monthly, and group differences were compared using the total score (0–10 points).

### Body Composition Analysis

Following a 4 h fasting period (with ad libitum access to water), body composition was assessed using a Small Animal Body Composition Analyzer (Minispec LF50, Bruker). Each mouse was gently placed in a dedicated detection mold to ensure proper positioning and comfort. Three consecutive scans were performed for each animal, and the results were recorded for further analysis.

### Glucose Tolerance Test

Following a 16 h fasting period (with ad libitum access to water), mice were transferred to clean cages. Baseline FBG levels (0 min) were measured. After a 30 min acclimatization period, an intraperitoneal glucose challenge (20% glucose solution, 0.01 mL g^−1^ body weight) was administered. Blood glucose levels were then monitored at 15, 30, 60, 90, and 120 min post‐injection.

### Grip Strength Test

At 22 and 24 months of age, forelimb muscle strength was evaluated using a tensile force tester (47200, Ugo Basile). Mice were carefully positioned on a horizontal testing grid and allowed to grasp firmly with their forepaws. A trained experimenter applied gradual backward traction along the longitudinal axis until the grip was released. Peak force values (in grams) were automatically recorded by the device, with three consecutive trials per animal at 1 min intervals to minimize fatigue.

### Rotarod Endurance Test

Motor endurance was assessed using a computer‐controlled rotarod apparatus (SA 102, Sansbio, China) under standardized conditions. The protocol included three consecutive trials with the following parameters: rod inclination of 15°, rotation speed of 20 rpm, and electrical stimulation (0.3 mA) to maintain motivation. Each trial continued until exhaustion, defined as the inability to maintain position for > 5 consecutive seconds, with a maximum cutoff time of 50 min per trial. The latency to exhaustion was automatically recorded for each trial, with 30 min rest periods between trials to allow recovery.

### Enzyme‐Linked Immunosorbent Assay

For each well, add 100 µL of the sample to be tested or a standard solution of varying concentrations. For blank wells, add 100 µL of Diluent A. Seal the plate with a new adhesive sealing film and incubate at room temperature for 2 h at 100–300 rpm on a plate shaker. After incubation, wash each well with 300 µL of wash buffer, allowing a 15–30 s interval between each injection and aspiration. Repeat the washing step five times. Following the final wash, carefully invert the plate and tap it firmly on absorbent paper to remove residual liquid, ensuring the wells are thoroughly drained. Next, dilute the detection antibody with Diluent B to its working concentration. Add 100 µL of the 1 × detection antibody working solution to each well, reseal the plate with a fresh sealing film, and incubate at room temperature for 1 h at 100–300 rpm. Wash the plate five times again. Then, dilute SA‐HRP in Diluent B to its working concentration. Add 100 µL of the 1× SA‐HRP working solution to each well, reseal the plate, and incubate at room temperature for 30 min at 100–300 rpm. Wash the plate five times once more. Add 90 µL of TMB substrate solution to each well, reseal the plate, and incubate in the dark at room temperature for color development. Finally, add 50 µL of stop solution to each well to terminate the reaction. Measure the absorbance at 450 nm within 10 min after termination.

### Histomorphological Staining

Tissue samples were fixed in 4% paraformaldehyde, processed through graded ethanol dehydration, and embedded in paraffin for sectioning. For CD11c immunofluorescence staining, antigen retrieval was performed using EDTA buffer and microwave irradiation, followed by blocking with 3% BSA and incubation with anti‐CD11c primary antibody at 4 °C overnight. After labeling with enzyme‐conjugated secondary antibody, nuclei were counterstained with DAPI, and slides were mounted with anti‐fade medium for fluorescence microscopy (Nikon ECLIPSE Ti2 Series, Nikon). Parallel sections were processed for TUNEL apoptosis detection, which involved dewaxing, rehydration, permeabilization, and incubation with the TUNEL reaction mixture (37 °C, 1 h in the dark). Apoptotic cells were identified by red fluorescence. For muscle fiber typing, frozen sections were stained with antibodies against MHC 2b (glycolytic fibers) and MHC 1 (oxidative fibers) at a 1:200 dilution, followed by nuclear counterstaining with Hoechst 33342. Conventional histological examination was performed on paraffin sections after standard H&E staining, with imaging conducted using an Upright Biological Microscope (Nikon Eclipse Ni‐U, Nikon). Histomorphological staining reagents are listed in Table S2 (Supporting Information).

### *β*‐Galactosidase Staining

MEF cells were plated in 6‐well culture plates at an appropriate density. After a 24 h incubation period to allow cell attachment, the culture medium was aspirated and replaced with 4% paraformaldehyde fixative for 15 min at room temperature. Following three washes with PBS, cells were incubated with freshly prepared Senescence‐associated β‐galactosidase (SA‐β‐gal) staining solution (pH 6.0) at 37 °C in a CO_2_‐free environment for 36 h. Senescent cells were identified by the development of distinct blue‐green cytoplasmic staining, observed under brightfield microscopy (Nikon Eclipse Ni‐U, Nikon).

### CCK‐8 Assay for Toxicity Analysis

Cells were plated in 96‐well culture plates at a density of 5 × 10^3^ cells well^−1^. After a 24 h incubation to allow cell attachment, cultures were treated with various concentrations (0, 2, 5, 10, 15, 30, 60, 120, 240, and 480 µg mL^−1^) of MSNs, SMSNs, or SeMSNs for 24 h. Then, 10 µL of CCK‐8 reagent was added to each well, and the plates were incubated at 37 °C for 1 h. Absorbance was measured at 450 nm using a microplate reader (Spark Multifunctional Microplate Detector, TECAN). Cell survival rates were calculated as (OD treatment/OD control) × 100%.

### Western Blot Detection

Total protein was extracted from cultured cells and mouse tissues using RIPA lysis buffer containing protease inhibitors. Protein concentrations were determined by the Bradford assay (Coomassie Brilliant Blue G‐250) with BSA as the standard. Equal amounts of protein were separated by SDS‐PAGE (8–12% gels) and electrophoretically transferred to PVDF membranes. After blocking with 5% non‐fat milk in PBST for 1 h at room temperature, membranes were incubated overnight at 4 °C with primary antibodies. After three washes with PBST, membranes were probed with HRP‐conjugated secondary antibodies (1:50 000) for 1 h at room temperature. Protein bands were detected using enhanced chemiluminescence reagents (Azure Biosystems, USA) and imaged with a chemiluminescence imaging system. Band intensities were quantified using ImageJ software (NIH, USA). Antibody catalog numbers are provided in (Table S3, Supporting Information).

### Transcriptome Sequencing Analysis

Tissue samples (brain, kidney, and gastrocnemius muscle) were collected from both the senescence model and SeMSNs‐treated groups (*n* = 3 per group). RNA sequencing was performed by Shanghai Bohao Biotechnology Co., Ltd. using the Illumina platform. Bioinformatics analysis was conducted to identify DEGs with thresholds of |log2FC| > 1 and false discovery rate (FDR) < 0.05 using the DESeq2 package. Functional annotation was carried out through Kyoto Encyclopedia of Genes and Genomes (KEGG) pathway analyses implemented in ClusterProfiler (version 4.0). GSEA was performed using GSEA v4.4.0. Comparative analysis was conducted, and Venn diagrams revealed overlapping DEGs across tissue types.^[^ ^66^ ^]^

### *qPCR* Detection

Total RNA was isolated from the samples using the SevenFast Total RNA Extraction Kit. RNA quality and concentration were assessed by spectrophotometric measurement (NanoPhotometer N60, IMPLEN). First‐strand cDNA synthesis was performed using 1 µg of total RNA with the Evo M‐MLV Reverse Transcription Kit II. Quantitative PCR was carried out using ChamQ Universal SYBR qPCR Master Mix on a real‐time fluorescence quantitative PCR instrument (LightCycler 96 Instrument, Roche) with the following cycling parameters: 95 °C for 30 sec, followed by 40 cycles of 95 °C for 5 sec and 60 °C for 45 sec. All reactions were performed in technical triplicate, and melting curve analysis was used to confirm amplification specificity. The sequences are provided in Tables S4 and S5 (Supporting Information).

### *ROS* Detection

Intracellular ROS levels were quantified using the ROS Assay Kit. Briefly, cells were incubated with 10 µm 2′,7′‐dichlorodihydrofluorescein diacetate (DCFH‐DA) working solution in serum‐free medium at 37 °C for 30 min in the dark. After incubation, cells were washed three times with PBS to remove excess probe. Fluorescence intensity (excitation/emission: 488/525 nm) was immediately measured using a multifunctional microplate reader (Spark Multifunctional Microplate Detector, TECAN). Data were normalized to baseline fluorescence (F0) and expressed as ΔF/F0.

### Calcium Ion Content Detection

Intracellular Ca^2+^ levels were assessed using the Fluo‐4 AM Calcium Assay Kit. Cells were loaded with 5 µm Fluo‐4 AM working solution and incubated at 37 °C for 30 min under light‐protected conditions. After incubation, cells were washed twice with PBS to remove extracellular dye and then equilibrated for an additional 15 min to ensure complete de‐esterification of the AM ester. Fluorescence intensity (Ex/Em = 494/528 nm) was measured in real‐time using a multifunctional microplate reader (Spark Multifunctional Microplate Detector, TECAN). Data were normalized to baseline fluorescence (F0) and expressed as ΔF/F0.

### Dual‐Luciferase Reporter Gene Assay

To investigate NFATc2‐mediated transcriptional regulation of Sik1, dual‐luciferase reporter assays were performed in HEK293T cells. The wild‐type Sik1 promoter region (43 427 031  to 43 429 131 bp relative to TSS) was cloned into the pGL3‐Basic vector, and a mutant construct (pGL3‐Sik1‐mut) was generated by site‐directed mutagenesis of the predicted NFATc2 binding site. Cells were co‐transfected in triplicate with the following groups: 1) Group 1: 0.5 µg pGL3‐Basic + 0.2 µg pCMV‐NFATc2(human)‐3 × FLAG‐Neo + 0.02 µg pRL‐TK; 2) Group 2: 0.5 µg pGL3‐Sik1 + 0.2 µg pCMV‐NFATc2(human)‐3 × FLAG‐Neo + 0.02 µg pRL‐TK; 3) Group 3: 0.5 µg pGL3‐Sik1‐mut + 0.2 µg pCMV‐NFATc2(human)‐3 × FLAG‐Neo + 0.02 µg pRL‐TK. A negative control was included for each group. After 12 h, cells were lysed and analyzed using the Dual–Lumi Luciferase Assay System. Firefly luciferase activity was normalized to Renilla luciferase (pRL‐TK) and expressed as fold‐change relative to control (mean ± SD, *n* = 3 independent experiments).

### ChIP‐qPCR Experiment

To investigate NFATc2 binding to genomic DNA, ChIP assays were performed in 293 cells (1 × 10^7^ cells per experimental group). Cells were crosslinked with 1% formaldehyde for 10 min at room temperature, followed by quenching with glycine. After lysis in ChIP buffer, chromatin was sonicated (SCIENTZ‐IID, SCIENTZ) to generate fragments ranging from 200 to 1000 bp. Immunoprecipitation was carried out overnight at 4 °C using an anti‐NFATc2 antibody (1:100), with normal IgG as a negative control. After reversal of crosslinks and DNA purification, enrichment of target genomic regions was quantified by qPCR using ChamQ Universal SYBR qPCR Master Mix. Relative binding was calculated using the ΔΔCt method, normalized to input DNA, and expressed as fold enrichment over IgG control. The sequences are provided in Table S5 (Supporting Information).

### Immunofluorescence Detection of NFATc2 Nuclear Translocation

This study comprised three experimental groups: untreated control, H_2_O_2_‐treated, and H_2_O_2_ + SeMSNs‐treated. After treatment, cells were fixed with 4% paraformaldehyde for 15 min at room temperature and permeabilized with 0.1% Triton X‐100 for 10 min. After blocking with 5% BSA for 1 h, cells were incubated overnight at 4 °C with anti‐NFATc2 primary antibody (1:200). Alexa Fluor 647‐conjugated secondary antibody (1:500) was applied for 1 h at room temperature, followed by nuclear counterstaining with DAPI. Fluorescence images were acquired using a fluorescence confocal microscope (Nikon ECLIPSE Ti2 Series, Nikon) with excitation/emission wavelengths of 633/668 nm for NFATc2 and 358/461 nm for DAPI, maintaining consistent acquisition parameters across all groups.

### Tissue Distribution of Selenium

Six wild‐type mice were randomly assigned to a control group and a SeMSNs treatment group. The control group received intragastric administration of normal saline, while the treatment group was administered SeMSNs. On the seventh day post‐treatment, the hearts, livers, spleens, lungs, kidneys, brains, and muscles were excised, weighed, immediately frozen in liquid nitrogen, and stored at −80 °C for subsequent analysis. The sample was dissolved in concentrated nitric acid and subsequently subjected to microwave‐assisted digestion. Following digestion, the mixture was heated at 100 °C for 2 h to remove residual acidic components. Once cooled, the digested sample was quantitatively transferred and diluted to exactly 1 mL with deionized ultrapure water. Finally, the selenium content was determined using inductively coupled plasma mass spectrometry (ICP‐MS).

### Oil Red O Staining

Remove the culture medium and rinse the cells twice with PBS. Fix the cells with a fixative solution for 25 min, then discard the fixative. Wash the cells twice with distilled water, followed by an immersion in 60% isopropanol for 25 s. Discard the isopropanol and add Oil Red O staining solution. Stain the cells for 15 min, then remove the staining solution and rinse again with 60% isopropanol for 25 s before discarding. Wash the cells five times with distilled water. Next, apply Mayer's hematoxylin staining solution and counterstain the nuclei for 2 min. Discard the hematoxylin solution and rinse the cells five times with distilled water. Incubate the cells with Oil Red O buffer solution for 1 min, then discard the buffer solution. Finally, cover the cells with distilled water, observe under a microscope, and capture images.

## Conflict of Interest

The authors declare no conflict of interest.

## Supporting information



Supporting Information

## Data Availability

All data needed to support the conclusions are presented in the paper or the supplemental information. Additional data related to this paper may be requested from the authors.
