# Supplementary material for: Enhanced Selenium Supplement Extends Lifespan and Delays Multi‐Organs Aging by Regulating the Sik1 Pathway Through Maintaining Calcium Homeostasis
Source: Adv Sci (Weinh). 2025 Oct 2;12(47):e11813. doi: 10.1002/advs.202511813 (PMC12713047; doi:10.1002/advs.202511813)
Supplement: Supplementary file 1 — Supporting Information [file ADVS-12-e11813-s001.docx]

Supporting Information

The novel nano-selenium supplement extends lifespan and delays multi-organs aging by regulating the Sik1 pathway through maintaining calcium homeostasis

Yang Yu, Jintao Song, Mengjiao Guo, RuZe Ma, Mingyang Du, Zhe Xun, Xu Liu, RongXia Xu, Xiaochun Xie, Peilin Qi, Yujie Chen, Dan Shao, Chao Yang, * Liang Wang, * Xiaoyu Song, * and Difei Wang*


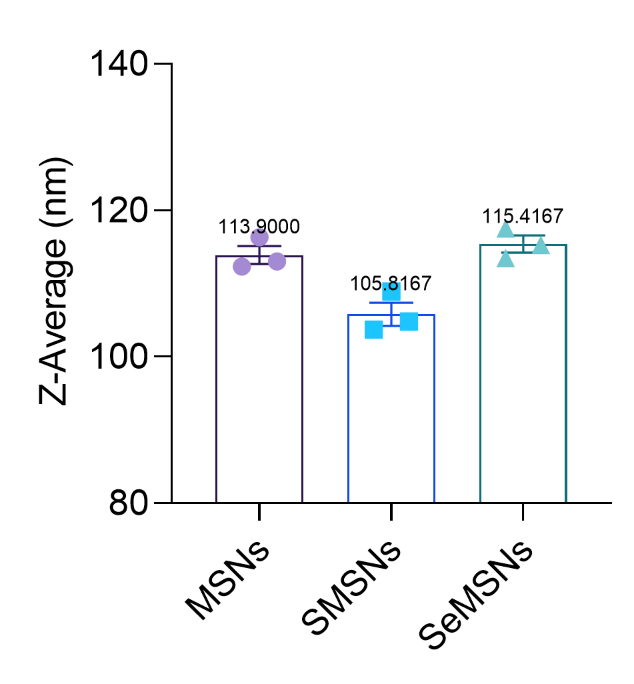


**Fig. S1. The hydrodynamic diameters of MSNs**, **SMSN**s **and SeMSN**s. The hydrodynamic diameters of MSNs, SMSNs and SeMSNs were measured by dynamic light scattering at 25℃ (n = 3 independent experiments). Samples were dispersed in deionized water (0.5 mg/mL) and sonicated for 10 min prior to analysis. The reported values are intensity-weighted averages from triplicate measurements.


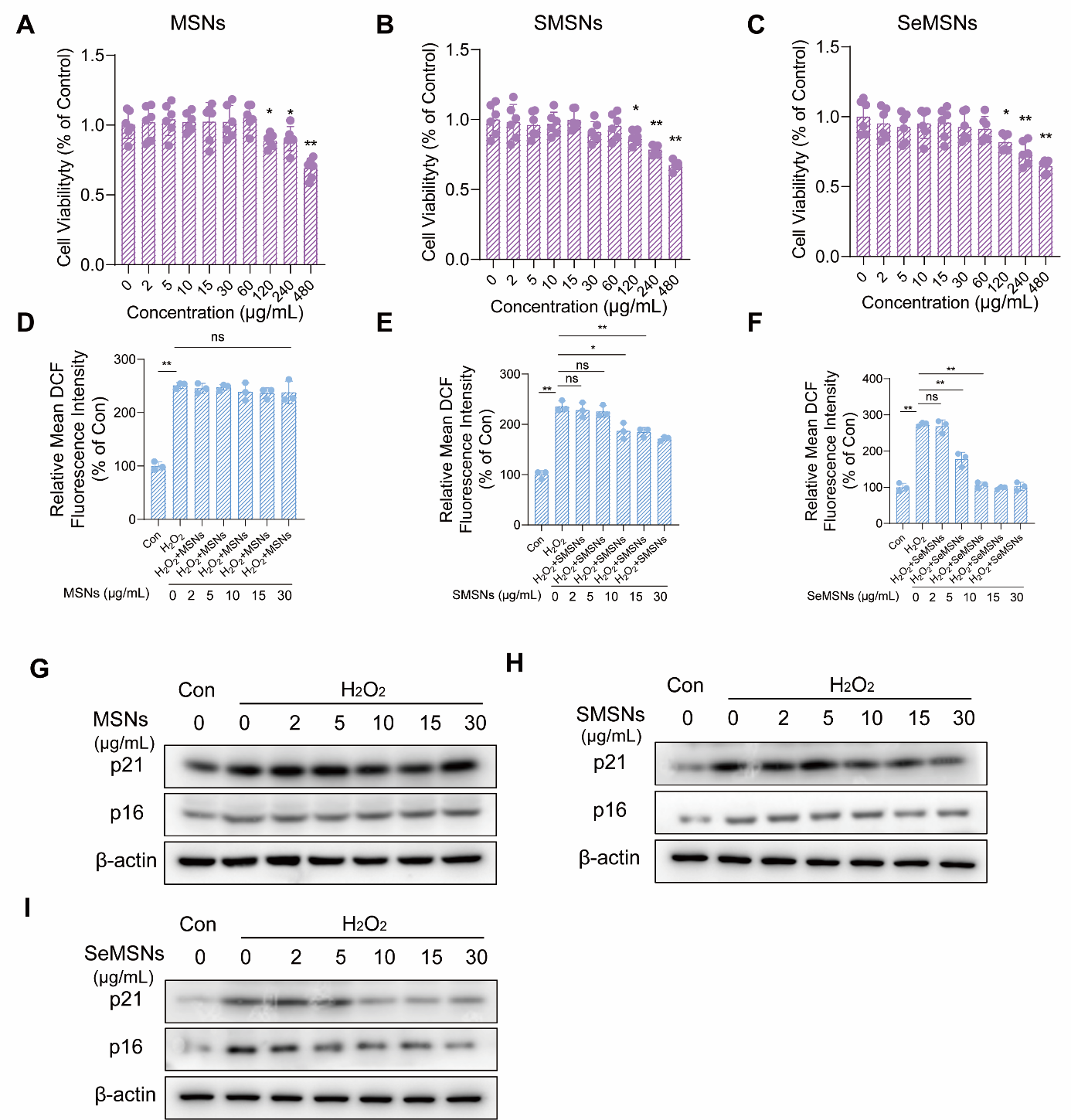


**Fig. S2. Evaluation of cytotoxicity, antioxidant effect and anti-aging effect of MSNs, SMSNs and SeMSNs at different concentrations.** (A)The cytotoxicity of MSNs (n = 6). (B)The cytotoxicity of SMSNs (n = 6). (C) The cytotoxicity of SeMSNs (n = 6). (D) Evaluation of antioxidant effects of MSNs (n = 3 independent experiments). (E) Evaluation of antioxidant effects of SMSNs (n = 3 independent experiments). (F) Evaluation of antioxidant effects of SeMSNs (n = 3 independent experiments). (G) Under oxidative stress conditions, the effects of different concentrations of MSNs on the expression of aging markers p21 and p16. (H) Under oxidative stress conditions, the effects of different concentrations of SMSNs on the expression of aging markers p21 and p16. (I) Under oxidative stress conditions, the effects of different concentrations of SeMSNs on the expression of aging markers p21 and p16. Data are presented as means ± SEM. Statistical differences were assessed via one-way ANOVA with Tukey’s multiple comparisons test. * denotes *p* < 0.05, ** denotes *p* < 0.01.


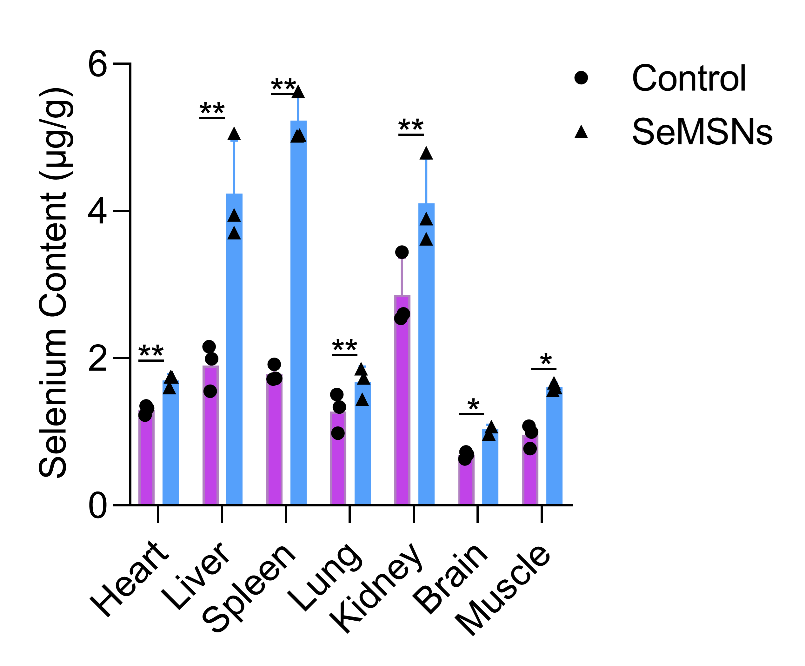


**Fig. S3. Tissue distribution determination of selenium**. The selenium content in the heart, liver, spleen, lung, kidney, brain and muscle tissues of mice was determined by ICP-MS (n = 3 mice per group). Data are presented as means ± SD. Statistical significance was done by Student’s t test. * Indicates *p* <0.05, * * indicates *p* <0.01.


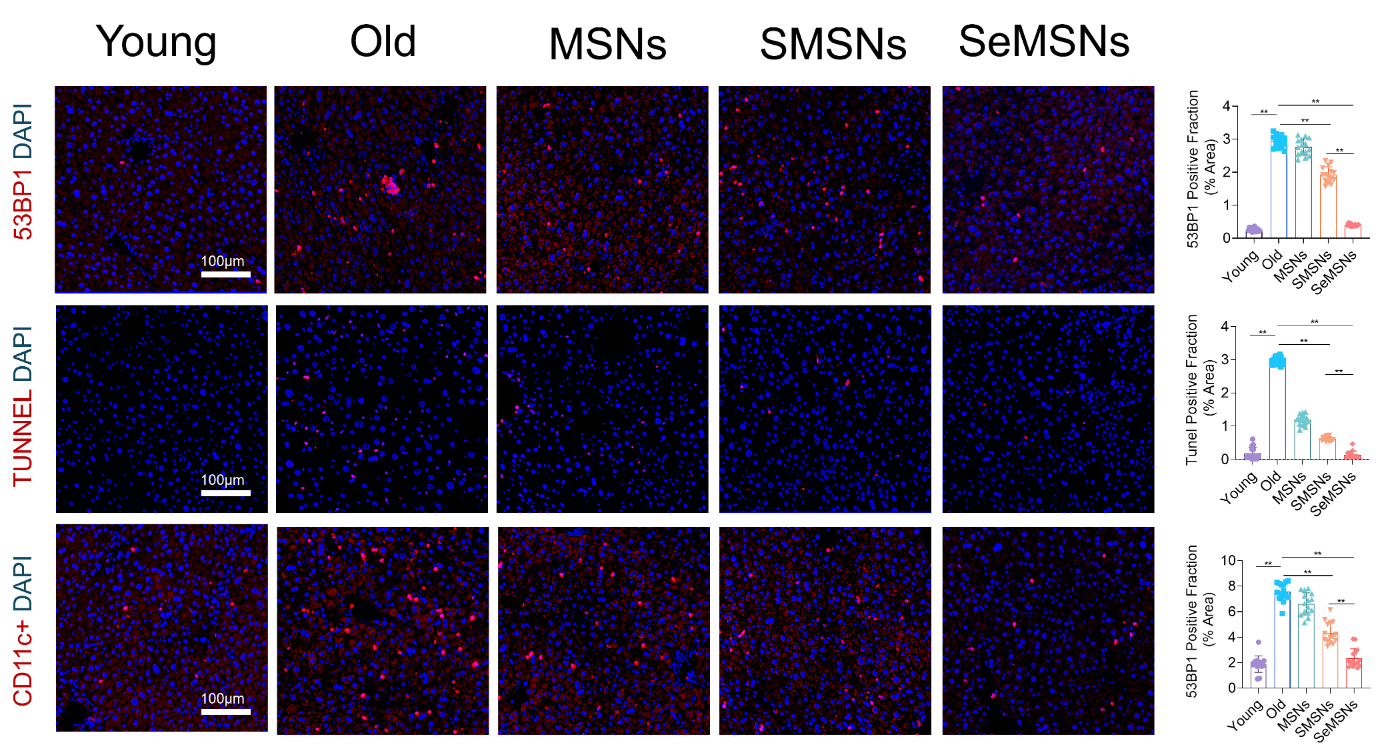


**Fig. S4. Multimodal analysis of liver tissues under the treatment of SeMSNs.** Multimodal analysis of renal tissues across five experimental groups: 53BP1 foci, TUNEL assay, and CD11c+ immune infiltration(5 fields per mouse were analyzed, n = 3 mice per group). All data are presented in the form of mean ± SEM. Statistical differences were assessed via one-way ANOVA with Tukey’s multiple comparisons test. * Indicates *p* <0.05, * * indicates *p* <0.01. Analyses performed using GraphPad Prism 9.


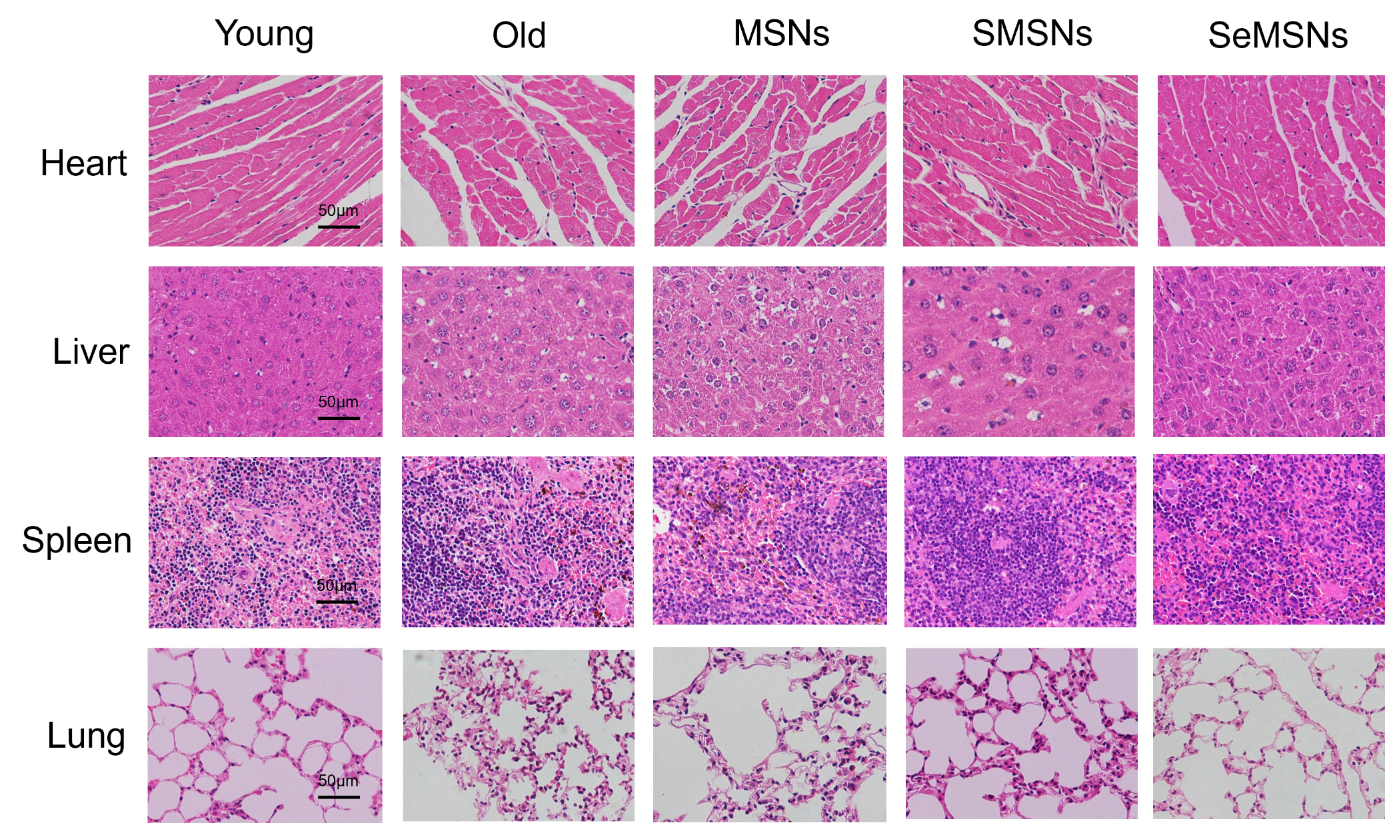


**Fig. S5. H&E staining of heart, liver, spleen, lung, and kidney samples from different groups of mice.** Young, Old, MSNs (mesoporous silica nanoparticles), SMSNs (disulfide-bond-bridged mesoporous silica nanoparticles), SeMSNs (diselenide-bond-bridged mesoporous silica nanoparticles).


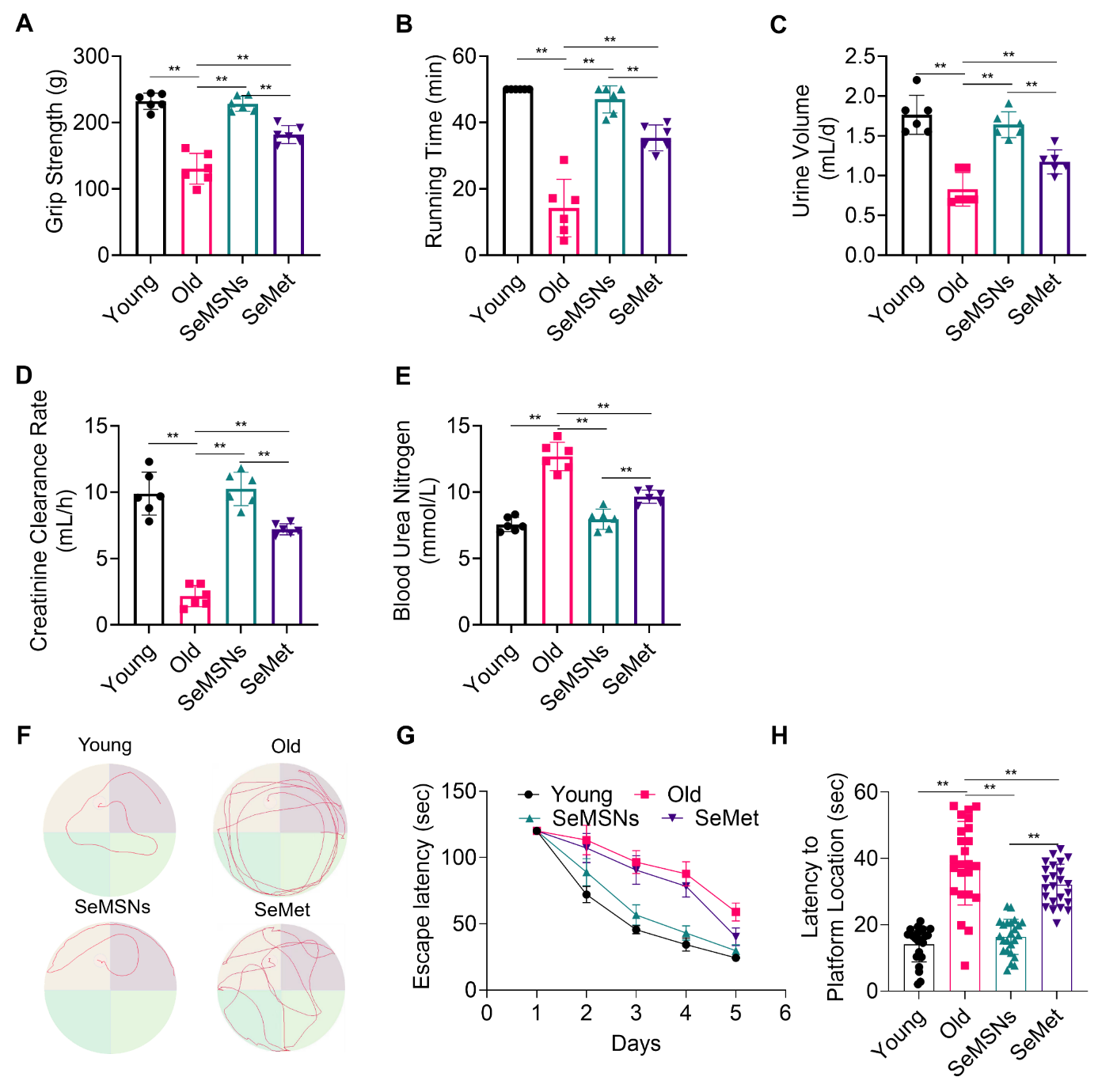


**Fig. S6. Comparison of the effectiveness of SeMSNs and SeMet in anti-aging.** (A) Comparison of grip strength among different groups of mice (n = 6 mice per group). (B) Comparison of running wheel time among different groups of mice (n = 6 mice per group). (C) Comparison of daily urine volume among different groups of mice (n = 6 mice per group). (D) Comparison of creatinine clearance rates among different groups of mice (n = 6 mice per group). (E) Comparison of plasma urea nitrogen levels in different groups of mice (n = 6 mice per group). (F) Pathway diagrams of water maze for mice in different groups. (G) Comparison of escape latency in different groups of mice (n = 6 mice per group). (H) Comparison of Latency to platform location in different groups of mice (Each mouse was tested once from each of the four quadrants, n = 6 mice per group). All data are presented in the form of mean ± SEM. Statistical differences were assessed via one-way ANOVA with Tukey’s multiple comparisons test. * Indicates *p* <0.05, * * indicates *p* <0.01. Analyses performed using GraphPad Prism 9.


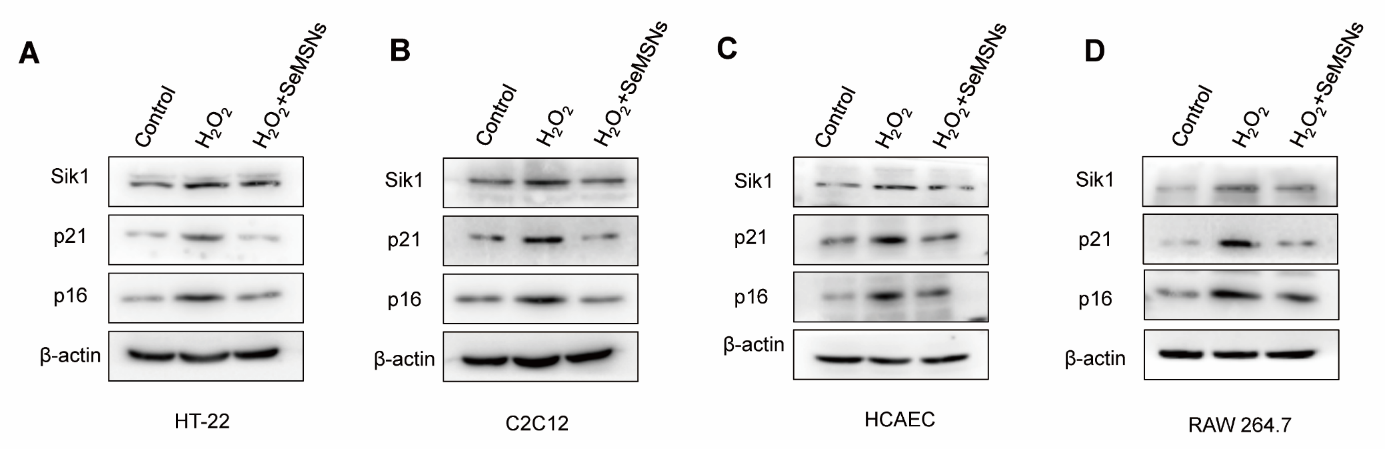


**Fig. S7. Modulation of Sik1, p21, and p16 in response to oxidative stress in diverse cell lines.** (A) Under oxidative stress conditions, the expression changes of Sik1 and the aging markers p21 and p16 in HT-22 cells. (B) Under oxidative stress conditions, the expression changes of Sik1 and the aging markers p21 and p16 in C2C12 myoblasts. (C) Under oxidative stress conditions, the expression changes of Sik1 and the aging markers p21 and p16 in HCAECs. (D) Under oxidative stress conditions, the expression changes of Sik1 and the aging markers p21 and p16 in RAW264.7 cells.


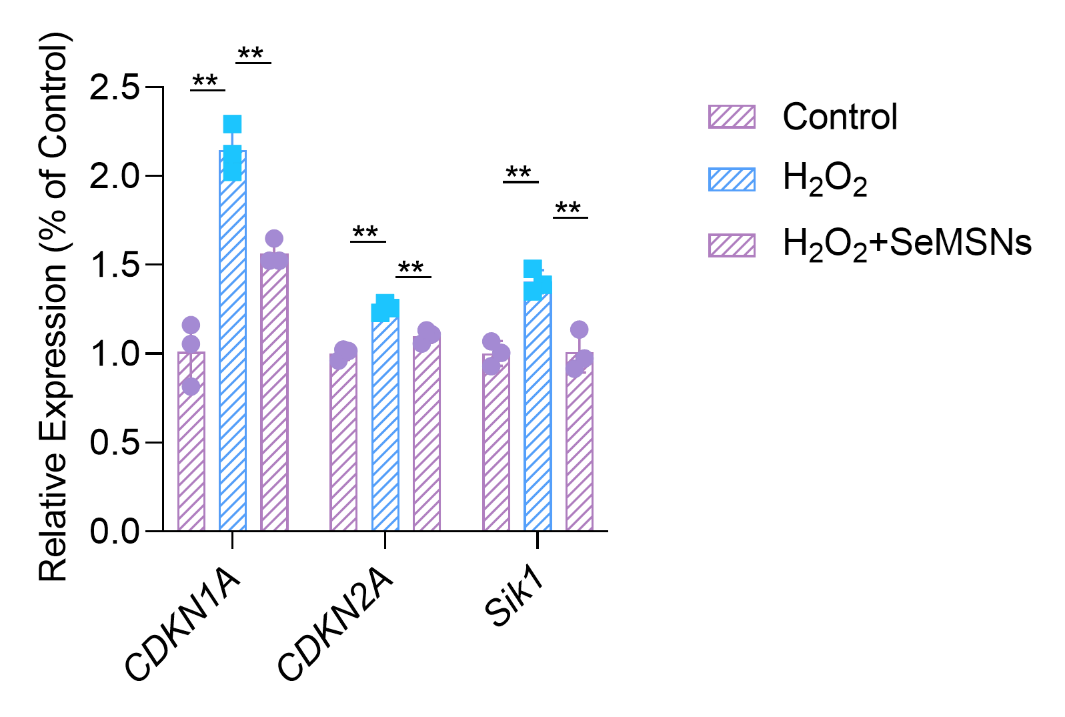


**Fig. S8. Effect of SeMSNs on H₂O₂-induced mRNA expression of CDKN1A, CDKN2A and SIK1 in HSF cells.** *CDKN1A*, *CDKN2A*, and *Sik1* represent the standard gene nomenclature for the protein products p21, p16, and Sik1, respectively (n = 3 independent experiments). All data are presented in the form of mean ± SD. Statistical significance was done by Student’s t test. * Indicates *p* <0.05, * * indicates *p* <0.01.


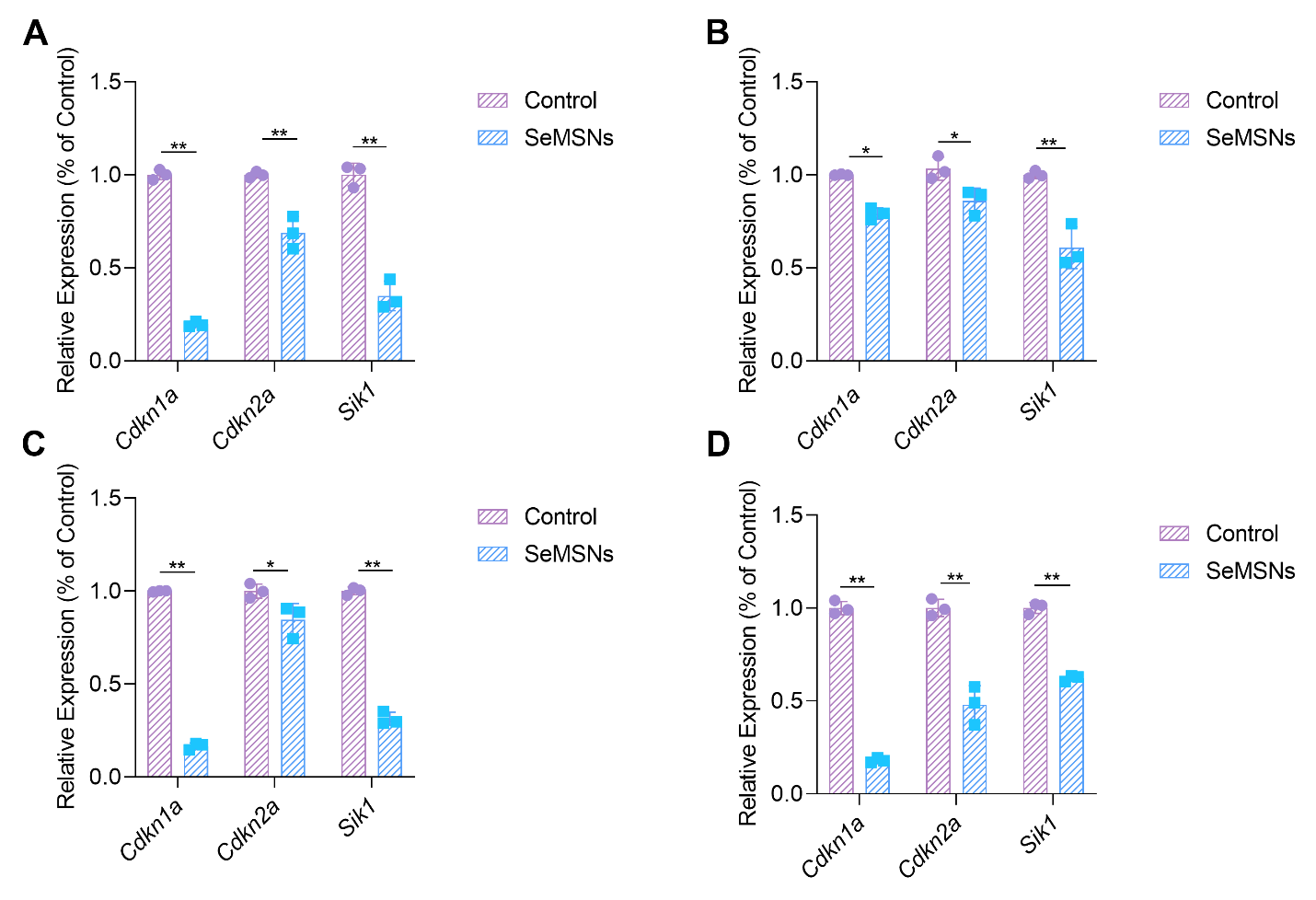


**Fig. S9. SeMSNs modulate the expression of *Cdkn1a*, *Cdkn2a*, and *Sik1* mRNA across multiple tissues in aged mice.** *Cdkn1a*, *Cdkn2a*, and *Sik1* represent the standard gene nomenclature for the protein products p21, p16, and Sik1, respectively. (A) Transcript levels of *Cdkn1a*, *Cdkn2a*, and *Sik1* in brain tissues were compared across two experimental groups (n = 3 independent experiments). (B) Transcript levels of *Cdkn1a*, *Cdkn2a*, and *Sik1* in kidney tissues were compared across two experimental groups (n = 3 independent experiments). (C) Transcript levels of *Cdkn1a*, *Cdkn2a*, and *Sik1* in GAS tissues were compared across two experimental groups (n = 3 independent experiments). (D) Transcript levels of *Cdkn1a*, *Cdkn2a*, and *Sik1* in liver tissues were compared across two experimental groups (n = 3 independent experiments). All data are presented in the form of mean ± SD. Statistical significance was done by Student’s t test. * Indicates *p* <0.05, * * indicates *p* <0.01.


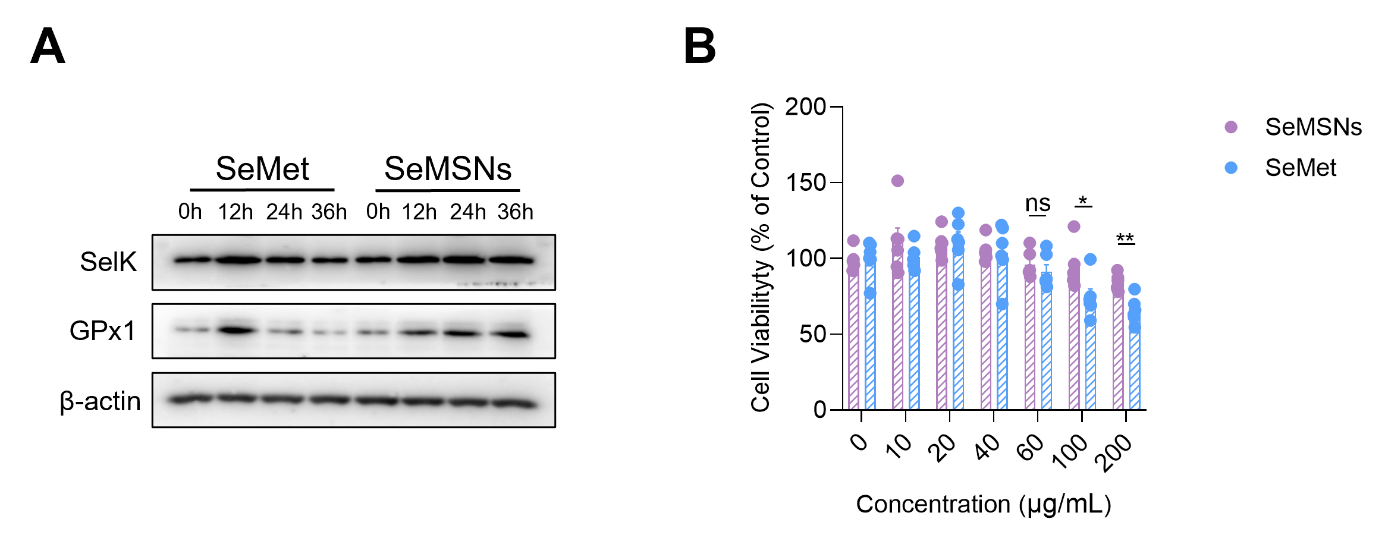


**Fig. S10. Comparing the selenium supplementation efficacy and biocompatibility between SeMet and SeMSNs.** (A) Time-dependent changes in SelK and GPx1 expression in adipocyte precursor cells after treatment with SeMet or SeMSNs (10 μg/mL; n = 3 independent experiments). (B) Comparative analysis of cell viability under treatment with SeMet or SeMSNs at varied concentrations (n = 6 independent experiments). Data are presented as means ± SEM. Statistical significance was done by Student’s t test. * denotes *p* < 0.05, ** denotes *p* < 0.01.

**Table S1. Baseline characteristics of study participants across young and old groups.​**

|  | Young individuals  (N = 30) | Old individuals  (N = 30) |
| --- | --- | --- |
| Age in years - mean (range) | 28.2 (24–35) | 70.8 (65–82) |
| Gender - M/F | 18/12 | 12/18 |
| Selenium (μg/mL) – mean ± SD | 32.18 ± 6.62 | 12.60 ± 3.24 |
| AST^a^ (U/L) – mean ± SD | 18.19 ± 6.55 | 51.27 ± 8.37 |
| ALT^b^ (U/L) – mean ± SD | 15.82 ± 5.56 | 48.06 ± 7.14 |
| TG^c^(mmol/L) – mean ± SD | 1.59 ± 0.11 | 1.76 ± 0.11 |
| TC^d^(mmol/L) – mean ± SD | 4.42 ± 0.30 | 5.23 ± 0.36 |
| CRP^e^(mg/L) – mean ± SD | 5.59 ± 0.42 | 26.02 ± 6.18 |
| FBG^f^(mmol/L) – mean ± SD | 4.52 ± 0.72 | 7.86 ± 1.09 |
| CRE^g^(μmol/L) – mean ± SD | 59.05 ± 6.39 | 86.92 ± 8.08 |
| SMI^h^ (kg/m^2^) – mean ± SD | 14.05 ± 4.10 | 4.37 ±0 .63 |
| HGS^i^ (kg) – mean ± SD | 34.27 ± 11.21 | 12.58 ± 4.77 |
| GS^j^(m/s) – mean ± SD | 0.93 ± 0.18 | 0.90 ± 0.24 |

a AST, glutamic oxaloacetic transaminase.

b ALT, alanine aminotransferase.

c TG, triglyceride.

d TC, total cholesterol.

e CRP, C-reactive protein.

f FBG, fasting blood glucose.

g CRE, creatinine.

h SMI, skeletal muscle index.

i HGS, hand-grip strength.

J GS, gait speed.

**Table S2. The** **reagents used in the experiment**

| **Reagent** | **Cat. No.** | **Company** |
| --- | --- | --- |
| H&E staining solution | G1005-500ML | Servicebio |
| TUNEL Apoptosis Detection Kit | G1502-50T | Servicebio |
| β-Galactosidase staining kit | G1580 | Solarbio |
| Reactive Oxygen Species Assay Kit | G1706-100T | Servicebio |
| Fluo-4 AM Fluorescence Calcium Ion Detection Kit | G1724-100T | Servicebio |
| Dual-Luciferase® Reporter Assay System | E1910 | Promega |

**Table S3. The antibodies used in the experiment.**

| **Antibody** | **Cat. No.** | **Company** |
| --- | --- | --- |
| Anti-p53 | 10442-1-AP | Proteintech |
| Anti-p21 | Ab109199 | Abcam |
| Anti-p16 | Ab51243 | Abcam |
| Anti-CD11c | GB11059-100 | Servicebio |
| Anti-Sik1 | 51045-1-AP | Proteintech |
| Anti-NFATc2 | 22023-1-AP | Proteintech |
| Anti-PP2A | 15882-1-AP | Proteintech |
| Anti-Perk | 20582-1-AP | Proteintech |
| Anti-Ire1 | 27528-1-AP | Proteintech |
| Anti-Atf6 | 24169-1-AP | Proteintech |
| Anti-SelK | 12561-1-AP | Proteintech |
| Anti-SelK | A20560 | ABclonal |
| Anti-GPx1 | 29329-1-AP | Proteintech |
| Anti- β-actin | 66009-1-Ig | Proteintech |
| Anti-GAPDH | 10494-1-AP | Proteintech |

**Table S4. The Primers used in the experiment (mouse)**

| **Primer** | **Sequence** |
| --- | --- |
| *Cdkn1a* forward primer | ACTTCCTCTGCCCTGCTGC |
| *Cdkn1a* reverse primer | GGTCTGCCTCCGTTTTCG |
| *Cdkn2a* forward primer | GATGGACGTTCAGGTGGCATA |
| *Cdkn2a* reverse primer | GCTTAGCAATGAAACTGCGAAGT |
| *Sik1* forward primer | CCAACCTGCCTACGCTGAG |
| *Sik1* reverse primer | GATCTGGGCTATGGTGATGCG |
| *Cacna1c* forward primer | GTTTCATTGTGTGTGGGGGC |
| *Cacna1c* reverse primer | AATGGAGCGCACTGAGTTCA |
| *Camk1* forward primer | GTGGAGAGCTGTTTGACCGA |
| *Camk1* reverse primer | GGTCGTGCAGGTACTTGACA |
| *Ryr1* forward primer | CGAGAAAATCGTGCGCAGAG |
| *Ryr1* reverse primer | AGTTGTACAGGGGCGTCATG |
| *Ryr3* forward primer | TGATGAGGCCTCCTGGATGA |
| *Ryr3* reverse primer | CACAGTCTTCACCGCCTTCT |
| *Itpr1* forward primer | CCCACCAATGCTGACATCCT |
| *Itpr1* reverse primer | ACCTCTTCCTCATCCTCCCC |
| *Stim1* forward primer | AGGAGATTGTGTCGCCCTTG |
| *Stim1* reverse primer | CGGATGCAGAGCAGAGAGAG |

**Table S5. The Primers used in the experiment (human)**

| **Primer** | **Sequence** |
| --- | --- |
| *CDKN1A* forward primer | AGGTGGACCTGGAGACTCTCAG |
| *CDKN1A* reverse primer | TCCTCTTGGAGAAGATCAGCCG |
| *CDKN2A* forward primer | CGGAAGGTCCCTCAGACATC |
| *CDKN2A* reverse primer | CCCTGTAGGACCTTCGGTGA |
| *Sik1* forward primer | CGCCATGTATAGTCGTCTCCC |
| *Sik1* reverse primer | GCCTTCAGCCCTTGAGTCAGT |
| *Sik1* promoter forward primer | GGGACTGAATGGTGAGTGTGATC |
| *Sik1* promoter reverse primer | CCAGCCTGAGCAAGAGAACAAG |
